# Supplementary material for: Emergence agitation in pediatrics after dexmedetomidine vs. sevoflurane anesthesia: A randomized controlled trial
Source: PLoS One. 2025 Oct 8;20(10):e0333576. doi: 10.1371/journal.pone.0333576 (PMC12507210; doi:10.1371/journal.pone.0333576)
Supplement: S1 Data — (PDF) [file pone.0333576.s001.pdf]

Study Protocol and Statistical Analysis Plan for Research Project:

"Emergence Agitation in Pediatrics after Dexmedetomidine vs. Sevoflurane Anesthesia: a randomized controlled trial"

Clinicaltrials.gov # NCT06482125

Conducted by Corry Quando Yahya, MD, Hori Hariyanto MD, PhD, Kohar Hari Santoso MD, PhD, Lucky Andriyanto MD, Arie Utariani MD, PhD, Bambang Pujo Semedi MD, PhD, Elizeus Hanindito MD, PhD

July 1, 2024

1. ABSTRACT

BACKGROUND

Emergence Agitation (EA) is a common occurrence in pediatric anesthesia. (1) The incidence has been reported to vary from 10% to as high as 80% after being exposed to inhalation anesthesia, particularly Sevoflurane. Maladaptive behaviors such as kicking, screaming, and thrashing are seen during agitation and contributes to postoperative complications such as tongue edema, re-bleeding of surgical wounds, falling out of bed, accidental removal of the intravenous line, bronchospasm and wound dehiscence. Anesthesia using Sevoflurane remain the main choice for cleft lip and cleft palate (CP) surgeries in children at the current moment. (1–3) As a result, majority of these children experience intense agitation after recovering from anesthesia.

OBJECTIVES

We propose a randomized controlled trial comparing emergence agitation in patients receiving Sevoflurane inhalation versus intravenous Dexmedetomidine infusion.

Primary Hypotheses:

The use of intravenous Dexmedetomidine reduces emergence agitation to children after anesthesia.

METHODS

All children enrolled to undergo an elective cleft lip or palate surgery at Pelita Harapan University and Siloam Hospital Lippo Village who fulfill the inclusion criteria will be selected.

2. SPECIFIC AIMS

Aim #1. To measure and compare emergence agitation between Sevoflurane and Dexmedetomidine group.
Aim #2. To measure and compare time to extubation between Sevoflurane and Dexmedetomidine group.
Aim #3: To measure and compare time to full recovery between Sevoflurane and Dexmedetomidine group.
Aim #4: Report any adverse events or side effects in both groups.

3. STUDY DESIGN

- A randomized controlled trial, single-blinded.
- Allocation: Simple Randomized (1:1) Patients are selected randomly using a computer-generated device to become a control arm (Sevoflurane) or treatment arm (Dexmedetomidine)

- Number of Arms: 2 (Sevoflurane vs. Dexmedetomidine)
- Masking: Single (Participant)

4. STUDY POPULATION

Children ages 3 months to 120 months (10 years) scheduled for an elective cleft lip or palate surgery in Pelita Harapan University and Siloam Hospital Village, Tangerang, Indonesia.

5. ELIGIBILITY TO PARTICIPATE ON THE STUDY

Minimum Age:	3 months
Maximum Age:	10 years
Sex:	All
Gender Based:	No
Accepts Healthy Volunteers:	Yes

Criteria:
Inclusion Criteria:
- Patients with weight ranging 5 kg - 25 kg
- Patients with American Society of Anesthesiologist (ASA) Physical Status Classification 1 and 2

Exclusion Criteria:
- Patients with any acquired congenital syndrome
- Patients who are actively taking anti-seizure medications and/or has been diagnosed with epilepsy
- Patients with functional and structural abnormalities of the heart, including arrythmias
- Patients with liver disease

6. RECRUITMENT

All children admitted to undergo an elective cleft lip or palate surgery between July 31, 2024 up to December 1, 2024.

7. DATA COLLECTION AND MEASURES

Data will be measured and collected starting July 31, 2024 up to December 1, 2024.

8. QUALITY ASSURANCE AND QUALITY CONTROL

Preoperative assessment and randomization of group type was performed one day before the operation.

9. RANDOMIZATION AND MASKING

A computer-generated random array sealed and envelope

method was employed to divide enrolled pediatric patients into two groups by simple randomization (1:1 ratio allocation). Experimental medication was prepared and provided by research staff who were not directly involved in patient care, while the surgeon, anesthesiologist and participating families were blinded to the medication distribution and group allocation.

On the day of surgery, the anesthesiologist responsible of the case was informed on each child's allocation and gave medications according to their designated groups. Agitation scale upon awakening and extubation time were recorded by a nurse anesthetist inside the operating room; while agitation monitoring every fifteen minutes were carried out by a different nurse in the recovery room without prior knowledge of the child's group allocation.

10. INTERVENTIONS

Anesthesia was administered using inhalation of 8% Sevoflurane in 100% oxygen until an intravenous line was secured. All patients were given Fentanyl 2 micrograms/kg, Propofol 3mg/kg and an oral endotracheal intubation of appropriate size was secured. After intubation, patients in Group DEX stopped receiving Sevoflurane. Dexmedetomidine infusion using an intravenous line was started at a loading dose of 1.5ug/kg for 10 minutes, followed by a maintenance dose of 1.5ug/kg/hour via a syringe pump while patients in Group SEVO continued receiving Sevoflurane at a concentration of 2-3 vol%.

All patients maintained spontaneous ventilation throughout the intraoperative period. Analgesia was supplemented with local anesthetic infiltration at the surgical site and intravenous Paracetamol 15mg/kg given 10 minutes before the end of surgical procedure. At the end of surgery, Dexmedetomidine infusion was stopped in group DEX and Sevoflurane inhalation was stopped in group SEVO: every one of them was extubated and moved to the post-anesthesia care unit for postoperative monitoring.

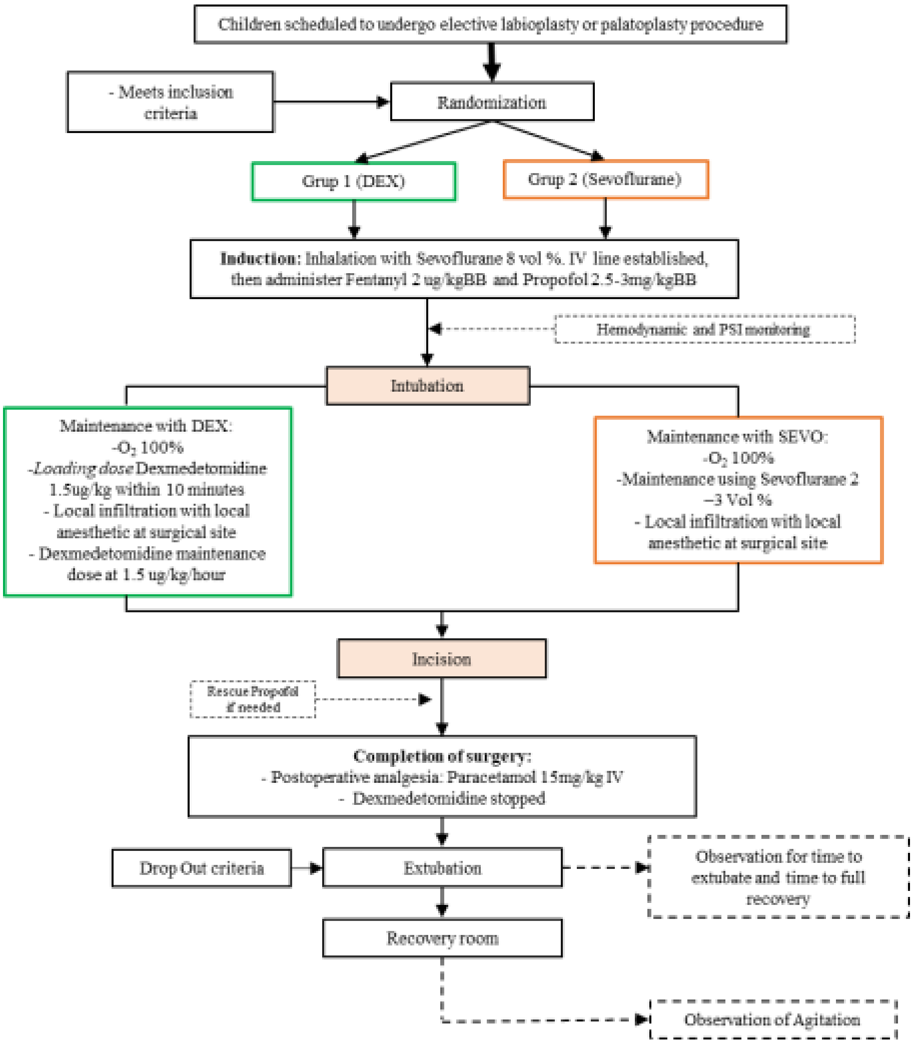

Fig 1. Flowchart of patient selection and grouping

11. SAFETY MONITORING

- ➢ Potential risks : bradycardia, hypotension, hypertension
- ➢ Alert values: Patients are continually monitored using standard monitoring which includes an electrocardiograph (ECG), non-invasive blood pressure and peripheral oxygen saturation (SpO2) which were recorded starting from induction and every 5 min, thereafter. Monitoring of anesthetic depth was ensured using SEDLine™ monitor, Masimo Corporation, Irvine, CA, USA to measure processed electroencephalograph or Patient State Index (PSI). Throughout the procedure, all patients retained their spontaneous respiration while maintaining their PSI value between 25-50.
- ➢ Reporting Procedures: written on a paper during research.

Preoperative anxiety status will be assessed by an anesthesiologist, the day before surgery. Time to extubation and agitation scale upon awakening will be recorded by the nurse anesthetist assisting the anesthesiologist on the day of operation. After transferring the child to the recovery room, another nurse will assess their agitation scale every 15 minutes using Cravero agitation scale. All these values are inputted on a computer after completion of anesthesia procedure, at the end of the day by a research staff who is not involved in patient care.

12. STATISTICAL POWER AND SAMPLE SIZE ESTIMATION

This study compares the incidence of agitation between the Sevoflurane group and the Dexmedetomidine group. This study is a categorical data, so the sample formula uses the formula:

$$n1 = n2 = \left(\frac{Z\alpha\sqrt{2PQ} + Z\beta\sqrt{P1Q1 + P2Q2}}{P1 - P2} \right)^2$$

Dexmedetomidine (p1) versus Sevoflurane (p2) anesthesia were analyzed with p1 being 10% and p2 being 40% with a power of 0.8 and alpha of 0.05. The power analysis study yielded sample size of 35 patients in each group. Considering 20% dropout case, we needed 42 cases per group and a total of 84 cases. Results were expressed as range (median), mean ± standard deviation, or number (%).

13. ANALYSIS PLAN

Missing and Incomplete data will be reported, if there are any.

14. DATA MANAGEMENT

Data confidentiality is ensured as all data are secured and locked in our institution. Access is only granted to the researchers involved.

15. TRIAL ADMINISTRATION - HOW INVESTIGATORS/STAFF WILL BE ORGANIZED

- The surgeon, anesthesiologist and participating families were blinded to the medication distribution and group allocation.
- After preoperative assessment and patient met the inclusion criteria, patients or the patient's family chose an envelope with a number inside it. The selected number later specified whether the patient was a control group or a treatment group, as randomized by the computer with 1:1 ratio allocation.

- Experimental medication was prepared and provided by research staff who were not directly involved in patient care for use the following day that surgery took place. On the day of operation, the anaesthesiologist responsible in anesthetizing the child will be informed of the child's group allocation in order to provide the appropriate medication according to their designated groups. The surgeon and recovery room nurse were not informed of the child's group allocation.

16. <u>TIMELINE</u>

- Research data gathering will be performed starting July 31, 2024 up to December 31, 2024.
- Data processing will start from January 1, 2025 to February 28, 2025.
- Research writing is expected to be completed at July 1, 2025.

References

1. Voepel-Lewis T, Malviya S, Tait AR. A Prospective Cohort Study of Emergence Agitation in the Pediatric Postanesthesia Care Unit. Anesth Analg [Internet]. 2003;96(6). Available from: https://journals.lww.com/anesthesia-analgesia/fulltext/2003/06000/a_prospective_cohort_study_of_emergence_agitation.16.aspx

2. Liu K, Liu C, Ulualp SO. Prevalence of Emergence Delirium in Children Undergoing Tonsillectomy and Adenoidectomy. Minervini G, editor. Anesthesiol Res Pract [Internet]. 2022;2022:1465999. Available from: https://doi.org/10.1155/2022/1465999

3. Moore AD, Anghelescu DL. Emergence Delirium in Pediatric Anesthesia. Paediatr Drugs. 2017 Feb;19(1):11–20.

4. Menser C, Smith H. Emergence Agitation and Delirium: Considerations for Epidemiology and Routine Monitoring in Pediatric Patients. Local Reg Anesth. 2020;13:73–83.

5. Reduque LL, Verghese ST. Paediatric emergence delirium. Contin Educ Anaesth Crit Care Pain [Internet]. 2013 Apr 1;13(2):39–41. Available from: https://doi.org/10.1093/bjaceaccp/mks051

6. Peng W, Zhang T. Dexmedetomidine decreases the emergence agitation in infant patients undergoing cleft palate repair surgery after general anesthesia. BMC Anesthesiol [Internet]. 2015;15(1):145. Available from: https://doi.org/10.1186/s12871-015-0124-7

7. Cravero J, Surgenor S, Whalen K. Emergence agitation in paediatric patients after sevoflurane anaesthesia and no surgery: a comparison with halothane. Paediatr Anaesth. 2000;10(4):419–24.

8. Andriyanto L, Utariani A, Hanindito E, Santoso KH, Puspita EA. Incidence of Emergence Agitation in Pediatric Patient after general anesthesia. Folia Medica Indones. 2019;55(March):25–9.

9. Desalu I, Adeyemo W, Akintimoye M, Adepoju A. Airway and respiratory complications in children undergoing cleft lip and palate repair. Ghana Med J. 2010 Mar;44(1):16–20.

10. Zhang J, Yin J, Li Y, Zhang Y, Bai Y, Yang H. Effect of dexmedetomidine on preventing perioperative respiratory adverse events in children: A systematic review and meta-analysis of randomized controlled trials. Exp Ther Med. 2023 Jun;25(6):286.

11. Li L, Zhang Z, Yao Z, Wang H, Wang H, An H, et al. The impact of laryngeal mask versus other airways on perioperative respiratory adverse events in

children: A systematic review and meta-analysis of randomized controlled trials. Int J Surg. 2019 Apr;64:40–8.

12. Kulkarni KR, Patil MR, Shirke AM, Jadhav SB. Perioperative respiratory complications in cleft lip and palate repairs: An audit of 1000 cases under "Smile Train Project". Indian J Anaesth. 2013 Nov;57(6):562–8.

13. Liu D, Pan L, Gao Y, Liu J, Li F, Li X, et al. Efficaciousness of dexmedetomidine in children undergoing cleft lip and palate repair: a systematic review and meta-analysis. BMJ Open. 2021 Aug;11(8):e046798.

14. Surana P, Parikh DA, Patkar GA, Tendolkar BA. A prospective randomized controlled double-blind trial to assess the effects of dexmedetomidine during cleft palate surgery. Korean J Anesthesiol [Internet]. 2017;70(6):633–41. Available from: http://europepmc.org/abstract/MED/29225747

15. Zhang X, Bai Y, Shi M, Ming S, Jin X, Xie Y. Effect of different administration and dosage of dexmedetomidine in the reduction of emergence agitation in children: a meta-analysis of randomized controlled trials with sequential trial analysis. Transl Pediatr [Internet]. 2021;10(4):929–57. Available from: http://europepmc.org/abstract/MED/34012842

16. Freriksen JJM, van der Zanden TM, Holsappel IGA, Molenbuur B, de Wildt SN. Best Evidence-Based Dosing Recommendations for Dexmedetomidine for Premedication and Procedural Sedation in Pediatrics: Outcome of a Risk-Benefit Analysis By the Dutch Pediatric Formulary. Paediatr Drugs. 2022 May;24(3):247–57.

17. Weerink MAS, Struys MMRF, Hannivoort LN, Barends CRM, Absalom AR, Colin P. Clinical Pharmacokinetics and Pharmacodynamics of Dexmedetomidine. Clin Pharmacokinet [Internet]. 2017;56(8):893–913. Available from: https://doi.org/10.1007/s40262-017-0507-7

18. Garcia PS, Kolesky SE, Jenkins A. General anesthetic actions on GABA(A) receptors. Curr Neuropharmacol. 2010 Mar;8(1):2–9.

19. Sakai EM, Connolly LA, Klauck JA. Inhalation anesthesiology and volatile liquid anesthetics: focus on isoflurane, desflurane, and sevoflurane. Pharmacotherapy. 2005 Dec;25(12):1773–88.

20. Torri G. Inhalation anesthetics: a review. Minerva Anestesiol. 2010 Mar;76(3):215–28.

21. Lee S-J, Sung T-Y. Emergence agitation: current knowledge and unresolved questions. Korean J Anesthesiol. 2020 Dec;73(6):471–85.

22. Kim JH. Mechanism of emergence agitation induced by sevoflurane anesthesia. Korean J Anesthesiol. 2011 Feb;60(2):73–4.

23. Mason KP. Paediatric emergence delirium: a comprehensive review and interpretation of the literature. Br J Anaesth. 2017 Mar;118(3):335–43.

24. Doerrfuss JI, Kramer S, Tafelski S, Spies CD, Wernecke K-D, Nachtigall I. Frequency, predictive factors and therapy of emergence delirium: data from a large observational

clinical trial in a broad spectrum of postoperative pediatric patients. Minerva Anestesiol. 2019 Jun;85(6):617–24.

25. Kayyal TA, Wolfswinkel EM, Weathers WM, Capehart SJ, Monson LA, Buchanan EP, et al. Treatment effects of dexmedetomidine and ketamine on postoperative analgesia after cleft palate repair. Craniomaxillofac Trauma Reconstr. 2014 Jun;7(2):131–8.

26. Nair S, Wolf A. Emergence delirium after paediatric anaesthesia: new strategies in avoidance and treatment. BJA Educ. 2018 Jan;18(1):30–3.

27. Mapelli J, Gandolfi D, Giuliani E, Casali S, Congi L, Barbieri A, et al. The effects of the general anesthetic sevoflurane on neurotransmission: an experimental and computational study. Sci Rep [Internet]. 2021;11(1):4335. Available from: https://doi.org/10.1038/s41598-021-83714-y

28. Massimini M, Ferrarelli F, Huber R, Esser SK, Singh H, Tononi G. Breakdown of cortical effective connectivity during sleep. Science. 2005 Sep;309(5744):2228–32.

29. Yang L, Ton H, Zhao R, Geron E, Li M, Dong Y, et al. Sevoflurane induces neuronal activation and behavioral hyperactivity in young mice. Sci Rep [Internet]. 2020;10(1):11226. Available from: https://doi.org/10.1038/s41598-020-66959-x

30. Goa KL, Noble S, Spencer CM. Sevoflurane in paediatric anaesthesia: a review. Paediatr Drugs. 1999;1(2):127–53.

31. Flacker JM, Lipsitz LA. Neural mechanisms of delirium: current hypotheses and evolving concepts. J Gerontol A Biol Sci Med Sci. 1999 Jun;54(6):B239-46.

32. Samuels ER, Szabadi E. Functional neuroanatomy of the noradrenergic locus coeruleus: its roles in the regulation of arousal and autonomic function part I: principles of functional organisation. Curr Neuropharmacol. 2008 Sep;6(3):235–53.

33. Toyoda Y, Zhu A, Kong F, Shan S, Zhao J, Wang N, et al. Structural basis of α1A-adrenergic receptor activation and recognition by an extracellular nanobody. Nat Commun. 2023;14(1):1–13.

34. Liu X, Li Y, Kang L, Wang Q. Recent Advances in the Clinical Value and Potential of Dexmedetomidine. J Inflamm Res. 2021;14:7507–27.

35. Coeckelenbergh S, Doria S, Patricio D, Perrin L, Engelman E, Rodriguez A, et al. Effect of dexmedetomidine on Nociception Level Index-guided remifentanil antinociception: A randomised controlled trial. Eur J Anaesthesiol. 2021 May;38(5):524–33.

36. Sottas CE, Anderson BJ. Dexmedetomidine: the new all-in-one drug in paediatric anaesthesia? Curr Opin Anaesthesiol. 2017 Aug;30(4):441–51.

37. Bailey CR. Dexmedetomidine in children – when should we be using it? Anaesthesia [Internet]. 2021;76(3):309–11. Available from: https://associationofanaesthetists-publications.onlinelibrary.wiley.com/doi/abs/10.1111/anae.15169

38. Gertler R, Brown HC, Mitchell DH, Silvius EN. Dexmedetomidine: a novel sedative-analgesic agent. Proc (Bayl Univ Med Cent). 2001 Jan;14(1):13–21.

39. Di M, Huang C, Chen F, Zeng R, Yu C, Shangguan W, et al. [Effect of single-dose

dexmedetomidine on recovery profiles after sevoflurane anesthesia with spontaneous respiration in pediatric patients undergoing cleft lip and palate repair]. Zhonghua Yi Xue Za Zhi [Internet]. 2014;94(19):1466–9. Available from: http://europepmc.org/abstract/MED/25143165

40. Mahmoud M, Barbi E, Mason KP. Dexmedetomidine: What's New for Pediatrics? A Narrative Review. J Clin Med. 2020 Aug;9(9).

41. Naaz S, Ozair E. Dexmedetomidine in current anaesthesia practice- a review. J Clin Diagn Res. 2014 Oct;8(10):GE01-4.

42. Su F, Hammer GB. Dexmedetomidine: pediatric pharmacology, clinical uses and safety. Expert Opin Drug Saf. 2011 Jan;10(1):55–66.

43. Denning S, Ng E, Wong Riff KWY. Anaesthesia for cleft lip and palate surgery. BJA Educ. 2021 Oct;21(10):384–9.

44. Machotta A. [Anesthetic management of pediatric cleft lip and cleft palate repair]. Anaesthesist. 2005 May;54(5):455–66.

45. Vyas T, Gupta P, Kumar S, Gupta R, Gupta T, Singh HP. Cleft of lip and palate: A review. J Fam Med Prim care. 2020 Jun;9(6):2621–5.

46. Fontanals M, Merritt G, Sierra P, Echaniz G. Anesthetic Considerations and Complications of Cleft Palate Repairs. What's New? Curr Anesthesiol Rep [Internet]. 2021;11(3):257–64. Available from: https://doi.org/10.1007/s40140-021-00460-7

47. Drover D, Ortega HR (Rick). Patient state index. Best Pract Res Clin Anaesthesiol [Internet]. 2006;20(1):121–8. Available from: https://www.sciencedirect.com/science/article/pii/S152168960500056X

48. Ricci Z, Robino C, Rufini P, Cumbo S, Cavallini S, Gobbi L, et al. Monitoring anesthesia depth with patient state index during pediatric surgery. Pediatr Anesth [Internet]. 2023 Oct 1;33(10):855–61. Available from: https://doi.org/10.1111/pan.14711

49. Sciusco A, Standing JF, Sheng Y, Raimondo P, Cinnella G, Dambrosio M. Effect of age on the performance of bispectral and entropy indices during sevoflurane pediatric anesthesia: a pharmacometric study. Pediatr Anesth [Internet]. 2017 Apr 1;27(4):399–408. Available from: https://doi.org/10.1111/pan.13086

50. Jang Y-E, Kim E-H, Lee J-H, Kim J-T, Kim H-S. Usefulness of bispectral index and patient state index during sevoflurane anesthesia in children: A prospective observational study. Medicine (Baltimore). 2022 Jul;101(30):e29925.

51. Soehle M, Ellerkmann RK, Grube M, Kuech M, Wirz S, Hoeft A, et al. Comparison between Bispectral Index and Patient State Index as Measures of the Electroencephalographic Effects of Sevoflurane. Anesthesiology [Internet]. 2008 Nov 1;109(5):799–805. Available from: https://doi.org/10.1097/ALN.0b013e3181895fd0

52. Jenkins BN, Fortier MA, Kaplan SH, Mayes LC, Kain ZN. Development of a short version of the modified Yale Preoperative Anxiety Scale. Anesth Analg. 2014 Sep;119(3):643–50.

53. Russell PSS, Mammen PM, Shankar SR, Viswanathan SA, Rebekah G, Russell S, et al. Pediatric Anesthesia Emergence Delirium Scale: A diagnostic meta-analysis. World J Clin Pediatr. 2022 Mar;11(2):196–205.

54. Kong H, Li M, Deng C-M, Wu Y-J, He S-T, Mu D-L. A comprehensive overview of clinical research on dexmedetomidine in the past 2 decades: A bibliometric analysis. Front Pharmacol. 2023;14:1043956.

55. Mahmoud M, Mason KP. Dexmedetomidine: review, update, and future considerations of paediatric perioperative and periprocedural applications and limitations. Br J Anaesth [Internet]. 2015 Aug 1;115(2):171–82. Available from: https://doi.org/10.1093/bja/aev226

56. Prayunanto E, Widyastuti Y, Sari D. The association of modified Yale perioperative anxiety scale and pediatric anesthesia behavior on postoperative emergence delirium in children: A prospective cohort study. Bali J Anesthesiol [Internet]. 2023;7(2). Available from: https://journals.lww.com/bjoa/fulltext/2023/07020/the_association_of_modified_yale_perioperative.6.aspx
