# Supplementary material for: Emergence agitation in pediatrics after dexmedetomidine vs. sevoflurane anesthesia: A randomized controlled trial
Source: PLoS One. 2025 Oct 8;20(10):e0333576. doi: 10.1371/journal.pone.0333576 (PMC12507210; doi:10.1371/journal.pone.0333576)
Supplement: S3 Data — (PDF) [file pone.0333576.s003.pdf]

PROPOSAL PENELITIAN

PERBANDINGAN KEJADIAN AGITASI PASCA ANESTESI INTRAVENA DEXMEDETOMIDINE DENGAN ANESTESI INHALASI SEVOFLURANE PADA OPERASI BIBIR SUMBING DAN CELAH LANGIT

CORRY QUANDO YAHYA

PROGRAM STUDI SUB SPESIALIS
ANESTESIOLOGI & TERAPI INTENSIF
FAKULTAS KEDOKTERAN UNIVERSITAS AIRLANGGA
RUMAH SAKIT UMUM PUSAT DR SOETOMO
SURABAYA
2024

PROPOSAL PENELITIAN

PERBANDINGAN KEJADIAN AGITASI PASCA ANESTESI INTRAVENA DEXMEDETOMIDINE DENGAN ANESTESI INHALASI SEVOFLURANE PADA OPERASI BIBIR SUMBING DAN CELAH LANGIT

Oleh :

Corry Quando Yahya
NIM. 605231002

PROGRAM STUDI SUB SPESIALIS
ANESTESIOLOGI & TERAPI INTENSIF
FAKULTAS KEDOKTERAN UNIVERSITAS AIRLANGGA
RUMAH SAKIT UMUM PUSAT DR SOETOMO
SURABAYA
2024

LEMBAR PENGESAHAN

PERBANDINGAN KEJADIAN AGITASI PASCA ANESTESI INTRAVENA DEXMEDETOMIDINE DENGAN ANESTESI INHALASI SEVOFLURANE PADA OPERASI BIBIR SUMBING DAN CELAH LANGIT

Usulan Penelitian ini Telah Disetujui untuk Diuji pada tanggal 5 Juni 2024

Penulis:
Corry Quando Yahya
NIM. 605231002

Pembimbing I	Pembimbing II
**Dr. Kohar Hari Santoso, dr., SpAn-TI., Subsp.An.Ped., Subsp TI**	**Lucky Andriyanto, dr., SpAn-TI Subsp. An.Ped., Subsp. TI**
**NIP. 19611203 198802 1 001**	**NIP. 19790201 201012 1 002**

Pembimbing III

Dr. Windhu Purnomo dr., M.S.
NIP. 19790201 201012 1 002

Mengetahui,

KPS Sp2 Anestesiologi & Terapi Intensif

Fakultas Kedokteran Universitas Airlangga

Prof. Dr. Nancy Margarita Rehatta, dr., SpAn-TI., Subsp. N.An., Subsp. M.N.
NIP. 19501003 202105 6 201

DAFTAR ISI

HALAMAN SAMPUL i
HALAMAN SAMPUL DALAM ii
LEMBAR PENGESAHAN iii
DAFTAR ISI iv
DAFTAR TABEL vi
DAFTAR GAMBAR vii
DAFTAR LAMPIRAN viii
DAFTAR SINGKATAN ix

BAB 1 PENDAHULUAN 1
1.1 Latar Belakang 1
1.2 Rumusan Masalah 3
1.3 Tujuan Penelitian 3
1.3.1 Tujuan umum 3
1.3.2 Tujuan khusus 3
1.4 Manfaat Penelitian 3
1.4.1 Manfaat teoritis 4
1.4.2 Manfaat praktis 4

BAB 2 TINJAUAN PUSTAKA 5
2.1 Agitasi pasca Anestesi 5
2.2 *Emergence Agitation dan Sevoflurane* 6
2.3 Locus Coereleus sebagai Target Anestesi Intravena 8
2.4 Mekanisme Kerja Dexmedetomidine 9
2.5 Dexmedetomidine dan Agitasi 10
2.6 Farmakokinetik Dexmedetomidine 11
2.7 Farmakodinamik Dexmedetomidine 12
2.8 Teknik Pembiusan Operasi Bibir Sumbing dan Celah Langit 13
2.9 Pemantauan Kedalaman Anestesi dengan *Patient State Index* (PSI) 14
2.10 Pengukuran Skala Agitasi 15

BAB 3 KERANGKA KONSEPTUAL DAN HIPOTESIS PENELITIAN 17
3.1 Kerangka Konsep 17
3.2 Narasi Penjelasan Kerangka Konsep 18
3.3 Hipotesis Penelitian 21

BAB 4 METODE PENELITIAN 22
4.1 Rancangan Penelitian 22
4.1.1 Jenis dan rancangan penelitian 22
4.2 Populasi, Besar Sampel dan Teknik Pengambilan Sampel 23
4.2.1 Populasi target 23

4.2.2 Populasi terjangkau 23
4.2.3 Sampel penelitian 23
4.2.4 Kriteria inklusi 23
4.2.5 Kriteria eksklusi 23
4.2.6 Kriteria *drop-out* 24
4.2.7 Perkiraan besar sampel 24
4.2.8 Cara pengambilan sampel 25
4.2.9 Prosedur penelitian 25
4.3 Variabel Penelitian dan Definisi Operasional 27
4.3.1 Variabel bebas 27
4.3.2 Variabel tergantung 27
4.3.3 Variabel perancu 28
4.3.4 Definisi operasional 28
4.4 Materi Penelitian 29
4.5 Instrumen Penelitian 29
4.5.1 SEDLine™ monitor, Masimo Corporation, Irvine, CA, USA 29
4.5.2 *Modified Yale Pre-operative Anxiety Scale-Short Form (*mYPAS-SF)..29
4.5.3 Syinge pump 29
4.5.4 Waktu ekstubasi 29
4.5.5 Waktu pilih sadar 29
4.5.6 Skala agitasi 30
4.6 Lokasi dan Waktu Penelitian 30
4.7 Kerangka Operasional Penelitian 31
4.8 Pengelolaan dan Analisis Data 31
4.9 Risiko Penelitian 32
4.10 Etika Penelitian 32

DAFTAR PUSTAKA 34

LAMPIRAN 39

DAFTAR TABEL

Tabel 2.1 Skala Cravero 16
Tabel 4.1 Definisi operasional 28

DAFTAR GAMBAR

Gambar 3.1 Diagram kerangka konseptual..17
Gambar 4.1 Kerangka operasional penelitian..31

DAFTAR LAMPIRAN

Lampiran 1. Lembar Penjelasan Penelitian..39
Lampiran 2. Lembar Persetujuan Partisipasi Penelitian..43
Lampiran 3. Lembar Persetujuan untuk Publikasi..43
Lampiran 4. Organisasi Peneliti 44
Lampiran 5. Lembar Penelitian 47
Lampiran 6. *Dummy Table*...51
Lampiran 7. Rincian Biaya Penelitian..53

DAFTAR SINGKATAN

ASA : *American Society of Anesthesiologist*

ASI : Air susu ibu

DEX : Dexmedetomidine

EA : *Emergence Agitation*

EE : *Electroencephalography*

GABA : Gamma-aminobutyric acid

NMDA : N-Methyl-D-Aspartate

VLPO : Preoptik lateral ventral

MnPN : Preoptik median

SSP : Sususan saraf pusat

LC : Locus Coeruleus

LMA : *Laryngeal Mask Airway*

NE : Norepinephrine

PSI : *Patient State Index*

α2AR : Alpha-2 Agonis reseptor

PRAE : *Perioperative Respiratory Adverse Events*

BAB 1

PENDAHULUAN

1.1 Latar Belakang

Agitasi pasca anestesi atau *Emergence Agitation* (EA) merupakan kejadian yang sering ditemukan pada pembiusan pediatrik. (1) Pada umumnya, pembedahan pada bibir sumbing dan celah langit memiliki skala nyeri yang tinggi sehingga penggunaan opioid dipadukan dengan pajanan konsentrasi anestesi inhalasi tinggi merupakan modalitas utama yang digunakan untuk memfasilitasi pembedahan. Hingga saat ini, anestesi inhalasi dengan Sevoflurane masih menjadi pilihan utama yang digunakan pada pembiusan pediatrik untuk operasi bibir sumbing dan celah langit. (1–3) Akibatnya, mayoritas dari anak mengalami agitasi hebat atau *Emergence Agitation* (EA) ketika pulih dari anestesi. (4)

Perilaku membahayakan seperti berteriak histeris, melengkungkan punggung, menendang dan menarik dapat menyebabkan komplikasi pasca operasi berupa pembengkakan lidah, pendarahan, jatuh dari tempat tidur, tercabutnya infus secara tidak sengaja, bronkospasme dan *wound dehiscence* atau luka terbuka. (5) Kejadian agitasi pasca anestesi inhalasi dilaporkan sebanyak 10 hingga 80%. (6,7) Penelitian yang dilakukan pada 105 pasien anak di Rumah Sakit Umum dr. Soetomo oleh Lucky et. al menunjukkan bahwa agitasi pasca anestesi terjadi pada 40% anak yang mendapatkan anestesi umum menggunakan anestesi inhalasi Sevoflurane. (8)

Oleh sebab itu, dibutuhkan obat anestesi yang aman tanpa efek samping yang dapat berdampak buruk. Selama ini, pembiusan untuk operasi bibir sumbing dan celah langit dilaksanakan pada anak mulai dari usia 3 bulan hingga usia sekolah

dan sering dikaitkan dengan berbagai masalah terkait jalan napas. (9) Insiden kritis yang dijumpai melibatkan jalan napas dan mayoritas timbul pada fase pemulihan dari anestesi. (10)(11) Adapun audit terhadap 1000 operasi bibir sumbing dan celah langit menemukan bahwa komplikasi pasca operasi terjadi pada 2.4% kasus bibir sumbing; 8.7% pada operasi celah langit dan 0.2% kejadian mortalitas yang disebabkan hipoksia. (12)

Obat anestesi yang ideal untuk pediatri seyogyanya memiliki efek anxiolitik, simpatolitik dan analgetik tanpa efek depresi napas atau agitasi pasca pembiusan. Properti dari obat tersebut dimiliki oleh Dexmedetomidine (DEX). (13,14) Dexmedetomidine merupakan agonis reseptor $\alpha 2$ sangat selektif dengan rasio reseptor $\alpha 2$: $\alpha 1$ 1620: 1. Berbeda dari propofol dan benzodiazepin, DEX tidak bekerja pada reseptor *gamma-aminobutyric acid* (GABA). (15) Efek hipnotik DEX dimediasi oleh aktivasi reseptor presinaptik dan postsinaptik $\alpha 2$ sentral pada locus coeruleus sehingga menyerupai tidur fisiologis dan tidur dalam. (6,16,17)

Efek sedasi yang dihasilkan DEX merupakan suatu keunikan yang belum didapati pada obat-obatan sedatif lainnya. Kemampuan DEX untuk mengurangi agitasi tanpa mengganggu pusat pernapasan pasca anestesi merupakan suatu keunggulan tersendiri sehingga penelitian ini bertujuan untuk menganalisis kejadian agitasi pasca anestesi total intravena Dexmedetomidine dan membandingkannya dengan anestesi inhalasi Sevoflurane pada anak yang menjalani operasi bibir sumbing dan celah langit.

1.2 Rumusan Masalah

Apakah terdapat perbedaan kejadian agitasi pasca anestesi anestesi intravena Dexmedetomidine dibandingkan dengan anestesi inhalasi Sevoflurane pada pasien pediatri yang menjalani operasi bibir sumbing dan celah langit?

1.3 Tujuan Penelitian

1.3.1 Tujuan umum

Menganalisis kejadian agitasi pasca anestesi antara anestesi intravena Dexmedetomidine dibandingkan dengan anestesi inhalasi Sevoflurane pada pasien pediatri yang menjalani operasi bibir sumbing dan celah langit.

1.3.2 Tujuan khusus

1. Menganalisis kejadian agitasi pasca anestesi pada anestesi intravena Dexmedetomidine.
2. Menganalisis kejadian agitasi pasca anestesi pada anestesi inhalasi Sevoflurane.
3. Menganalisis perbedaan kejadian agitasi pasca anestesi antara anestesi intravena Dexmedetomidine dibandingkan dengan anestesi inhalasi Sevoflurane.

1.4 Manfaat Penelitian

1. Hasil analisis penelitian ini akan bermanfaat sebagai dasar ilmiah penggunaan intravena Dexmedetomidine untuk pasien pediatri di kamar operasi
2. Pengembangan teknik anestesi pediatri dengan penggunaan metode intravena Demedetomidine dapat memicu penelitian lanjutan

3. Hasil penelitian ini dapat dijadikan sebagai dasar penelitian lebih lanjut mengenai manajemen agitasi pasca pembiusan

1.4.1 Manfaat teoritis

Dosis Dexmedetomidine yang ditemukan pada penelitian ini dapat dijadikan sebagai formula untuk penggunaan total anestesi intravena pada populasi pediatri.

1.4.2 Manfaat praktis

Hasil penelitian ini dapat digunakan dalam panduan praktis klinis (PPK) anestesi pediatri.

BAB 2

TINJAUAN PUSTAKA

2.1 Agitasi Pasca Anestesi

Agen anestesi mengubah perilaku neuron dengan berinteraksi langsung melalui kanal ion susunan saraf pusat. Anestesi umum bertindak dengan meningkatkan sinyal penghambatan GABA reseptor atau memblokir sinyal yang memiliki sifat eksitasi. Saat ini, terdapat lima obat anestesi inhalasi dan lima anestesi intravena yang digunakan untuk menginduksi atau mempertahankan anestesi umum. (18)

Mayoritas obat anestesi memiliki pusat kerja pada reseptor GABA, namun terdapat beberapa yang bekerja dengan memblokir aksi glutamat, neurotransmitter rangsang utama di otak. Propofol dan Barbiturat merupakan obat anestesi yang bekerja melalui reseptor GABA-A. (18) Anestesi inhalasi telah digunakan sejak tahun 1846 dengan penggunaan Eter pertama kali. Anestesi inhalasi bekerja untuk menekan neurotransmisi jalur eksitasi yang melibatkan asetilkolin (reseptor muskarinik dan nikotinik), glutamat (reseptor NMDA), dan serotonin (reseptor 5-HT) dalam sistem susunan saraf pusat (SSP) dan meningkatkan sinyal inhibisi pada reseptor GABA untuk memberikan tingkat sedasi yang memadai. (19,20)

Faktor risiko timbulnya agitasi pasca anestesi adalah usia, jenis kelamin laki-laki, jenis operasi, operasi darurat, penggunaan anestesi inhalasi dengan koefisien partisi darah-gas rendah, durasi operasi yang lama, pemberian antikolinergik, premedikasi dengan benzodiazepin, nyeri pasca operasi dan adanya perangkat invasif. (21) Mekanisme patofisiologi agitasi pasca anestesi belum

diketahui secara tepat. Namun, pada anak-anak, penyebab agitasi yang diusulkan termasuk tingkat kecemasan yang tinggi mengenai operasi, lingkungan baru dan pemisahan dari orang tua. Hal ini menyebabkan peningkatan aktivitas simpatik dan perpanjangan keadaan tereksitasi selama pemulihan anestesi. (22)

Penjelasan yang diusulkan untuk timbulnya agitasi pasca anestesi akibat anestesi inhalasi adalah perbedaan tingkat pemulihan diferensial dalam fungsi otak, akibat *clearance* anestesi inhalasi dari sistem saraf pusat yang bervariasi. Fungsi audisi dan motorik pulih terlebih dahulu diikuti oleh konektivitas talamokortikal dan sensorik, sedangkan pemulihan sistem talamoregulasi subkortikal tertunda sehingga akan berdampak pada disfungsi dalam integrasi informasi kortikal, yang dapat menyebabkan kebingungan atau keadaan gelisah saat pulih dari anestesi umum. (21,22)

Meskipun agitasi pasca anestesi bersifat sementara dan mereda seiring dengan waktu (15 - 45 menit pasca pembiusan) (23), beberapa obat seperti pemberian Propofol, Clonidine, Midazolam dan Dexmedetomidine telah dilaporkan efektif untuk menurunkan angka kejadian agitasi pasca anestesi. (10,15,24–26)

2.2 *Emergence Agitation* dan Sevoflurane

Anestesi volatil telah banyak digunakan untuk induksi dan pemeliharaan anestesi selama lebih dari 170 tahun, namun mekanisme kerja obat tersebut tetap sulit dipahami. Masing masing obat anestesi menginduksi keadaan anestesi melalui target molekuler dan sirkuit saraf yang berbeda dan dapat dinilai dari gelombang *electroencephalograhy* (EEG). (27) Dengan demikian, keadaan otak dapat

dimanifestasikan oleh perubahan karakteristik dalam aktivitas EEG. Induksi anestesi dengan Sevoflurane terkait dengan aktivitas elektroensefalografi seperti kejang dan telah dilaporkan dalam studi observasional (28), tetapi tidak jelas apakah aktivitas elektroensefalografi (EEG) epileptiform ini sama dengan gambaran EEG selama agitasi.

Patofisiologi terjadinya agitasi telah diteliti oleh Mapelli et. al dimana Ia menemukan mekanisme anestesi Sevoflurane yang berpusat pada daerah serebellum. Sevoflurane mengubah neurotransmisi dengan meningkatkan penghambatan neuron GABA dan mengurangi aktivitas NMDA glutamatergik. Perubahan ini menyebabkan penurunan neurotransmisi yang signifikan dalam sel granula serebelar (GrCs) setelah aktivasi berulang oleh *excitatory mossy fibers* (mfs) dan mengubah rangsangan intrinsik GrCs yang mempromosikan generasi potensial aksi. (27) Beberapa bagian dari neuron berada dalam kondisi eksitasi bahkan pada saat anestesia berlangsung. Namun, berkurangnya respons GrCs yang ditimbulkan oleh mfs mendukung gagasan bahwa Sevoflurane menyebabkan hambatan komunikasi antar saraf tanpa membungkam aktivitas neuron intrasel. (29)

Adapun beberapa penelitian pada hewan menunjukkan bahwa anestesi inhalasi Sevoflurane dapat menimbulkan agitasi melalui aktivasi neuronal pada beberapa bagian tertentu didalam otak. Penelitian oleh Yang *et al,* menemukan bahwa paparan anestesi inhalasi Sevoflurane dapat menimbulkan aktivasi protein C-Fos, sebuah protein onkogen yang bersifat stimulatori. (29) Daerah otak yang terlibat pada timbulnya agitasi adalah daerah korteks prefrontal, talamus dan basal ganglia. Hal ini terjadi akibat perubahan pada sejumlah besar neurotransmiter: yang

paling sering dan paling banyak ditandai adalah pengurangan fungsi kolinergik dan peningkatan fungsi dopaminergik dan gabaergik, meskipun perubahan di hampir semua sistem neurotransmitter : serotoninergik, noradrenergik, glutaminergik, histaminergik telah ditemukan. (4,5,30,31)

Teori 'diferensial klirens' menggambarkan bagaimana Sevoflurane memiliki waktu klirens dari sistem saraf pusat yang berbeda sehingga waktu pemulihan fungsi kognitif akan bervariasi. Oleh karena itu, agitasi menandakan ketidakseimbangan antara kondisi seseorang 'terbangun' dan seseorang 'sadar'. (21,23) Hal ini menyebabkan pasien bangun, membuka mata, namun tidak dapat memahami informasi dari lingkungan dan memberikan respons dan interaksi yang sesuai.

2.3 Locus Coeruleus sebagai Target Anestesi Intravena

Lokus coeruleus (LC) adalah struktur otak belakang yang merupakan sumber utama distribusi norepinefrin (NE) ke daerah korteks dan lobus frontalis. Struktur ini merupakan situs utama dalam sintesis norepinefrin otak yang terlibat dalam proses kewaspadaan, atensi maupun stres. LC dan area tubuh yang dipengaruhi oleh norepinefrin dinamakan sebagai sistem lokus coeruleus-noradrenergik. (32,33)

Sistem LC noradrenergik memiliki proyeksi neuron yang sangat ekstensif. Serabut saraf tersebut memiliki proyeksi ke sebagian besar korteks serebral, neuron kolinergik pada daerah basal otak, neuron dari talamus ke korteks, pengendalian fungsi otonom melalui proyeksi langsung ke sumsum tulang belakang dan inti otonom, dan proyeksi penghambatan substansial untuk neuron GABAergik pada daerah basal otak depan dan area preoptik ventrolateral. (32,34)

Dengan demikian, aktivasi LC akan menghasilkan pola kompleks aktivitas saraf di seluruh otak yang diamati sebagai peningkatan dalam respons atensi, kewaspadaan and peningkatan aktivitas simpatis seseorang. Sebaliknya, penghambatan pada system LC akan menyebabkan penurunan kesadaran yang menyerupai tidur fisiologis. (32)

Perkembangan ilmu telah menemukan reseptor α-2 adrenergik yang memiliki jumlah kepadatan tertinggi pada locus coeruleus, sebuah inti noradrenergik yang merupakan modulator penting untuk tingkat kewaspadaan. Disamping itu, Locus Coeruleus ditemukan memiliki jaras medulospinalis noradrenergik yang merupakan modulator penting dari neurotransmiter nosiseptif. (35,36) Penemuan ini menjadikan landasan dari penggunaan obat noradrenergik selektif sebagai agen hipnosis.

2.4 Mekanisme Kerja Dexmedetomidine

Dexmedetomidine (DEX) adalah obat dengan subklasifikasi imidazole yang memiliki senyawa mirip klonidin, namun lebih selektif, spesifik dan poten sebagai agonis α-2 adrenergik (α2:α1 1620:1). DEX mempunyai waktu kerja yang lebih pendek dengan waktu paruh 2 – 3 jam, dibandingkan dengan klonidin yang merupakan α-2 agonis sebagian dikarenakan α2:α1 = 220:1 ; dengan waktu paruh 12-24 jam. (17,37)

Efek fisiologis DEX dimediasi melalui stimulasi reseptor α-2 adrenergik postsinaps yang akan mengaktivasi protein G, sehingga menghasilkan umpan balik inhibitorik serta penurunan aktivitas adenylyl cyclase. Hal ini mengakibatkan penurunan cAMP intrasel dan aktivitas protein kinase yang bergantung terhadap

cAMP. Proses ini pada akhirnya akan memodifikasi translokasi ion dan konduksi membran. (17,37)

Mekanisme yang signifikan untuk menghambat reseptor α-2 adrenergik, adalah terjadinya hiperpolarisasi pada susunan sel syaraf pusat, dan masuknya protein ke dalam kanal kalium, sehingga mengurangi eksitasi. Selain itu, protein G0 yang teraktivasi juga dapat mengakibatkan berkurangnya konduktasi kalsium ke dalam sel melalui *voltage-gated ion channel* type N yang bekerja tidak dipengaruhi oleh cAMP dan fosforilasi protein, sehingga terjadi hambatan pelepasan neurotransmitter. (38)

2.5 Dexmedetomidine dan Agitasi

DEX menstimulasi tingkat parasimpatis dan inhibisi simpatis secara sentral dari locus ceruleus dan mengakibatkan penurunan aktivasi neuron noradrenergik dari Locus Coeruleus dan peningaktan aktivitas sistem neuron inhibitorik GABA, sehingga menimbulkan efek sedasi, serta anxiolisis atau antinosiseptif. DEX akan mengaktifkan reseptor α-2 adrenergik postsinaps pada pusat vasomotor meduler di otak dan sumsum tulang belakang, mengurangi norepinefrin dan menurunkan tingkat simpatis sentral, sehingga menghambat sinyal nyeri atau nosiseptif, serta mengurangi frekuensi nadi dan tekanan darah. (32,36)

Aktivasi reseptor α-2 adrenergik pada kornu dorsalis medulla spinalis akan menghambat pelepasan substansi P, menimbulkan efek analgesik primer dan potensiasi efek analgesik dari opioid. Mekanisme terjadinya efek analgesik dari obat α-2 agonis masih belum dapat sepenuhnya dijelaskan. Namun diperkirakan secara teoritis terdapat dua mekanisme yang berbeda untuk pengaruh terhadap

sinyal nyeri, yang pertama adalah pencegahan eksitasi syaraf, dan kedua adalah pencegahan penyebaran sinyal ke syaraf disekitarnya. Beberapa jaras persyarafan seperti sistem syaraf pusat supraspinal, medulla spinalis, serta reseptor α-2 adrenergik perifer, dapat memodulasi sinyal transmisi nosiseptif yang merupakan sinyal nyeri. Obat-obat pengurang rasa nyeri dapat bekerja pada lokasi ini. (35)

Interaksi dengan reseptor α-2 adrenergik pada sistem syaraf pusat dan medulla spinalis mengakibatkan efek fisiologis pada beberapa sistem organ, antara lain sedasi, ansiolisis, analgesi, penurunan MAC agen anestesi inhalasi, penurunan kadar renin dan vasopressin yang mengakibatkan diuresis, penurunan respon simpatis terhadap operasi, dan penurunan frekuensi nadi dan tekanan darah. (16,38)

2.6 Farmakokinetik Dexmedetomidine

Dexmedetomidine adalah obat yang memiliki ikatan protein yang kuat, dengan 94% berikat dengan Albumin dan Alpha-1 glikoprotein. DEX memiliki volume distribusi berkisar 1.31–2.46 L/kg (90–194 L) dan waktu paruh distribusi 6 menit. DEX mempunyai waktu kerja yang lebih pendek dengan waktu paruh 2 – 3 jam, dibandingkan dengan klonidin yang merupakan α-2 agonis sebagian dikarenakan α2:α1 = 220:1 ; dengan waktu paruh 12-24 jam. (17,37) Pemberian DEX secara oral memiliki efek *first-pass* dengan 16% bioavailabilitas. Analisis non-kompartemen menunjukkan eliminasi DEX adalah 0.6-0.7 L/menit dengan waktu paruh eliminasi 2.1-3.1 jam. (17,39)

Pada anak berusia lebih dari 1 bulan, DEX menunjukkan tingkat kemanjuran yang serupa dengan orang dewasa tanpa efek samping yang merugikan. Tingkat klirens DEX pada anak berusia 2 bulan hingga 6 tahun lebih tinggi (0,8-1,2

L/kg/jam) dibandingkan dengan anak yang lebih tua dan orang dewasa (0,6-0,7 L/kg/jam). Sebuah penelitian yang melibatkan 669 anak berumur 0,1 hingga 22,5 tahun menemukan dosis awal 0,5-1,0 ug/kg yang diberikan selama 10-20 menit efektif dalam memberikan sedasi tanpa menurunkan laju napas atau obstruksi jalan napas. Efek samping hemodinamik berupa bradikardia lebih sering terjadi pada anak yang lebih tua, namun tidak memerlukan intervensi farmakologis. (17)

Metabolisme DEX terjadi di hati. Pada pasien dengan gangguan hati, penurunan klirens dan fraksi dexmedetomidine yang tidak terikat akan lebih tinggi, sehingga efek klinis DEX akan lebih lama. Kemampuan ikatan plasma protein DEX pada paisen dengan gangguan hati ringan, sedang, dan berat adalah 87,9%, 86,0%, dan 82,0% dibandingkan dengan 89,8% pada subjek normal. Oleh sebab itu, dosis DEX perlu dikurangi sesuai dengan derajat gangguan hati. (17)

2.7 Farmakodinamik Dexmedetomidine

Sedasi dengan DEX mempromosikan tidur endogen sehingga menyerupai tidur alami. [49, 50]. Efek sedasi DEX dipengaruhi oleh konsentrasi plasma dengan konsentrasi plasma 0,2 dan 0,3 ng/mL akan menghasilkan sedasi dimana pasien tetap dapat dibangunkan dan memberikan respons yang sesuai. Sedasi dalam dimana pasien tidak dapat dibangunkan dan memberikan respons terjadi saat kadar konsentrasi plasma diatas 1,9 ng/mL. (17,40)

DEX menghasilkan respons hemodinamik bifasik yang khas: hipotensi pada konsentrasi plasma rendah dan hipertensi pada konsentrasi plasma tinggi. Administrasi bolus secara intravena akan menghasilkan konsentrasi plasma yang tinggi sehingga terjadi hipertensi dan bradikardia pada saat pemberian dosis awal. Sesudah beberapa menit, konsentrasi plasma DEX akan menurun, seiring dengan

aktivasi reseptor alpha-2 pada endotel vaskular dengan efek vasodilatasi. Bersamaan dengan aktivasi a2-adrenoreseptor prasinaps akan menghambat pelepasan katekolamin dan meningkatan aktivitas vagal sehingga terjadi fase hipotensi dengan penurunan 13-27% dari nilai tekanan darah awal. (40)

Dengan konsentrasi plasma terapeutik hingga 2,4 ng/mL, kejadian depresi pernapasan minimal dan respon ventilasi terhadap karbon dioksida tetap terjaga. Respons ventilasi hiperkapnik diketahui menurun seiring bertambahnya usia. Oleh karena itu, pasien usia lanjut lebih rentan mengalami depresi pernafasan dibandingkan dengan usia muda. (36,41)

Efek samping dari DEX termasuk hipotensi, hipertensi, mual, bradikardia, fibrilasi atrium, dan hipoksia. Ketika DEX diberikan bersamaan dengan obat penenang lainnya: agen hipnotik atau analgetik, efek sedatif menjadi sinergis dengan peningkatan risiko depresi ventilasi atau apnea. Disamping itu, limitasi penggunaan DEX terdapat pada pasien dengan kelainan jantung karena penggunaan dosis awal meningkatkan hipertensi paru dan sistemik yang signifikan sehingga DEX sebaiknya dihindari atau diberikan dengan dosis yang lebih rendah. (42)

Overdosis dapat menyebabkan blok atrioventrikular tingkat pertama atau kedua. Sebagian besar efek samping terjadi selama atau sebentar setelah pemberian obat dosis awal (*loading dose*). Dengan menghilangkan atau mengurangi dosis awal, efek samping dapat dikurangi. (16)

2.8 Teknik Pembiusan Operasi Bibir Sumbing dan Celah Langit

Perbaikan primer bibir sumbing dilakukan pada saat anak berusia diantara 6 minggu hingga 6 bulan agar dapat memungkinkan pertumbuhan pasien secara psikososial yang tepat. Jika sumbing perbaikan langit-langit diperlukan, maka

operasi akan dilakukan pada usia diantara 6 dan 12 bulan pada saat anak mulai berbicara atau membentuk fonasi. Mayoritas (61%) celah bibir dan celah langit hadir sebagai kelainan yang tidak memiliki sindrom dengan etiologi yang masih belum jelas. (43)

Anestesi dapat dipertahankan dengan menggunakan anestesi inhalasi atau anestesi intravena. Penambahan Remifentanil dapat mengurangi kebutuhan anestesi volatil dan propofol dan memungkinkan ventilasi terkontrol. Penggunaan relaksasi otot dapat diberikan atau tidak. Ventilasi spontan juga merupakan alternatif lain dalam pembedahan bibir sumbing dan celah langit. Waktu operasi bervariasi antara 1 jam hingga 4 jam. (44) Analgesia multimodal untuk prosedur bibir dan langit sumbing dapat diberikan secara enteral dan teknik anestesi lokal. Pilihan diantaranya adalah Acetaminophen oral (15-20 mg/kg), NSAID (Ibuprofen 5-10 mg/kg) atau Ketorolac (0.5mg/kg). Anestesi lokal dapat diberikan melalui infiltrasi Lidocaine dan Epinephrine atau Blok Supraorbital. (45,46)

2.9 Pemantauan Kedalaman Anestesi dengan *Patient State Index* (PSI)

Pemantauan kedalaman anestesi sangat penting selama tindakan. Pada umumnya, tingkat kedalaman anestesi diperkirakan berdasarkan tanda vital seperti denyut jantung, tekanan darah, atau gerakan sebagai respons terhadap rangsangan nyeri. Namun, parameter ini telah terbukti tidak dapat diandalkan dalam menilai kedalaman anestesi ataupun mencegah kesadaran intraoperatif. Anestesi yang terlalu dangkal menimbulkan komplikasi serius dengan gejala psikologis jangka panjang seperti kecemasan dan gangguan stres pascatrauma sedangkan anestesi yang terlalu dalam dapat menyebabkan perubahan hemodinamik. Oleh sebab itu,

pada penelitian ini, alat SedLine® sensor (Masimo Corporation; Irvine, CA, USA) menggunakan *Patient State Index* (PSI) untuk memantau kedalaman anestesi. (47,48)

Indeks keadaan pasien atau PSI adalah indeks turunan Electroensefalogram (EEG) yang dikembangkan untuk menilai kedalaman anestesi. PSI beroperasi pada skala 0 hingga 100, dengan angka yang lebih rendah mencerminkan tingkat anestesi yang lebih dalam. Nilai 0 mewakili tidak adanya aktivitas otak, dan 100 mewakili keadaan terjaga. Nilai kurang dari 25 mewakili keadaan hipnosis yang dalam. Nilai PSI antara 25 dan 50 mewakili anestesi umum yang memadai untuk operasi dan mencegah kesadaran di bawah anestesi. (49,50) Sebuah studi menunjukkan bahwa PSI mampu memberikan estimasi kedalaman anestesi yang memadai sama seperti pemantauan dengan alat *Bispectral index* (BIS). (51)

2.10 Pengukuran Skala Agitasi

Prosedur pembedahan daerah tenggorok, hidung, telinga dan mata telah terbukti meningkatkan risiko agitasi pasca anestesi dibandingkan dengan prosedur bedah urologi dan bedah umum lainnya. Pasien usia balita, terutama dalam rentang usia 2 hingga 5 tahun berada pada peningkatan risiko. (21) Kecemasan pra-operasi yang diukur dengan Skala Kecemasan Pra Operasi Yale (*Modified Yale Preoperative Anxiety scale*), menunjukkan adanya peningkatkan risiko agitasi pasca anestesi pada anak-anak yang mengalami kecemasan. (52)

Selanjutnya, kejadian dan derajat agitasi dapat diamati dan dinilai dengan menggunakan skala Cravero, yang sudah memiliki uji validitas. (4,53) Skala Cravero mengkategorikan lima tingkat kesadaran mulai dari "tidak ada respons

terhadap rangsangan" (Skor 1) hingga "perilaku meronta-ronta yang membutuhkan pengekangan" bahkan setelah upaya menenangkan secara aktif (Skor 4 atau 5). (Tabel 2.1)

Skala Cravero mengkategorikan lima tingkat kesadaran mulai dari "tidak ada respons" (Skor 1) hingga "perilaku meronta-ronta yang membutuhkan pengekangan" (Skor 5). Setelah upaya menenangkan aktif, pasien yang memiliki agitasi tinggi pada skala Cravero (Skor 4 atau 5) disebut sebagai agitasi pasca anestesi. (21)(4)

Tabel 2.1 Skala Cravero

Skala	Perilaku
1	Tidak memberikan respon pada stimulasi
2	Tertidur tenang. Anak memberikan respons saat diberikan stimulasi
3	Bangun dan memberikan respon kepada pengasuh
4	Menangis lebih dari 3 menit, tanpa perilaku kombatif
5	Menangis meraung, melakukan tindakan kombatif seperti mencabut infus, menendang dan tidak dapat ditenangkan oleh siapapun
1-3 = tidak delirium, 4-5 = delirium	

BAB 3

KERANGKA KONSEPTUAL DAN HIPOTESIS PENELITIAN

3.1 Kerangka Konsep

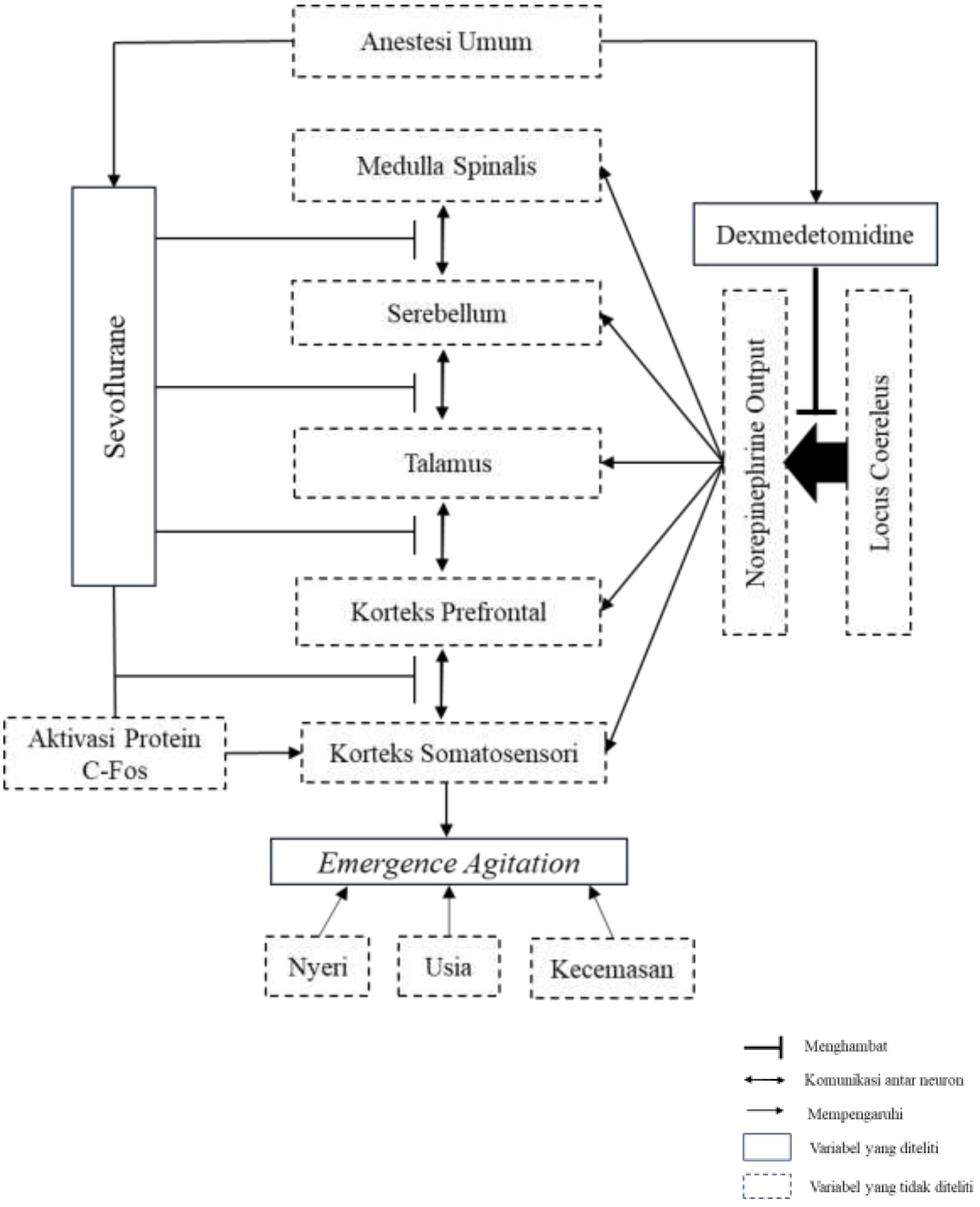

Gambar 3.1 Diagram kerangka konseptual

3.2 Narasi Penjelasan Kerangka Konsep

Mekanisme timbulnya agitasi masih diselidiki hingga hari ini. Adapun beberapa penelitian pada hewan menunjukkan bahwa anestesi inhalasi Sevoflurane dapat menimbulkan agitasi melalui aktivasi neuronal pada beberapa bagian tertentu didalam otak. Penelitian oleh Yang *et al,* menemukan bahwa paparan anestesi inhalasi Sevoflurane dapat menimbulkan aktivasi protein C-Fos, sebuah protein onkogen yang bersifat stimulatori. (29)

Protein C-Fos berfungsi sebagai faktor transkripsi dalam pengaturan gen dan digunakan sebagai penanda fungsional aktivitas neuron dan sirkuit saraf. Dalam kondisi normal, ekspresi kadar protein c-Fos rendah. Namun, melalui gambaran histopatologik imunofluoresens Yang et. al menemukan bahwa paparan anestesi inhalasi Sevofurane menyebabkan peningkatkan kadar C-Fos yang terpusatkan pada daerah korteks somatosensori sel otak tikus. Secara klinis, aktivasi neuronal pada Korteks Somatosensori menimbulkan perilaku hiperaktif yang ditandai oleh gerakan dan/atau hentakan keempat ekstremitas tikus. Temuan ini menunjukkan bahwa aktivasi neuron pada Korteks Somatosensori dapat berkontribusi pada kejadian perilaku kombatif yang sering diamati saat proses pemulihan dari Sevoflurane. (29)

Di sisi lain, Sevoflurane mengurangi neurotransmisi dengan peningkatkan GABA reseptor yang bersifat inhibitori dan mengurangi aktivitas NMDA glutamatergic yang bersifat eksitatori. Sevoflurane menghambat kemampuan komunikasi antar neuron tanpa meredam aktivitas neuronal intrasel sehingga target utama Sevoflurane sebenarnya berpusat pada daerah transmisi sinaptik antar sel dan efek 'tidak sadar' ditimbulkan oleh komunikasi sinaps yang terputus. (27)

Pemulihan dari efek Sevoflurane ditemukan berbeda pada setiap bagian otak sehingga pemulihan pada korteks tidak akan sama seperti pada daerah talamus maupun cerebelum. (21) Maka, efek klinis yang timbul akibat kecepatan pulih yang bervariasi adalah seseorang yang membuka mata dan menggerakan tangan dan kaki, tanpa menyadari sepenuhnya tindakan yang Ia diperbuat. Pada akhirnya, efek hipoaktivasi dan berkurangnya NMDA reseptor adalah munculnya halusinasi atau 'mimpi buruk' yang terwujud dengan efek klinis berupa delirium dan/atau agitasi yang dialami pasca paparan Sevoflurane. (27)

Hal ini berbeda dengan Dexmedetomidine. Efek Dexmedetomidine dimediasi melalui aktivasi reseptor α2 pra dan pasca sinaps pada Lokus Coeruleus. Lokus Coeruleus adalah inti dari Pons yang menghasilkan dan menyimpan neurotransmitter Norepinefrin terbanyak di otak. (54) Sebagai neurotransmitter, Norepinephrine memiliki peran untuk mempertahan keadaan mawas diri dan terjaga (sadar penuh). Maka, pengikatan Dexmedetomidine dengan Alpha 2 reseptor (α2AR) akan menghambat pelepasan Norepinefrin dan menghentikan propagasi neurotransmisi. Secara molekuler, efek yang ditimbulkan adalah ion kalium yang keluar dan penghambatan aliran masuk ion kalsium sehingga membuat hiperpolarisasi sel membran sel dan menurukan ambang ransangan sel neuron. Efek klinis yang ditemukan adalah sedasi yang menyerupai tidur alami, tanpa efek pada pusat pernapasan.

Selain sedasi, DEX memiliki efek dalam mengurangi rangsangan sistem saraf simpatik dan mengurangi respons stress. Kadar Norepinephrine yang berkurang berdampak pada penghambatan rangsangan saraf simpatik akibat penghambatan aktivitas adenilat siklase dan pengurangan siklik adenosin

monofosfat sehingga mengurangi konsentrasi katekolamin plasma, menstabilkan hemodinamik, mengurangi tekanan darah dan detak jantung. (55)

Efek unik lain yang dimiliki DEX adalah analgesia. DEX mengikat α2AR pada susunan saraf pusat untuk menghentikan transmisi sinyal nyeri; sedangkan DEX mengaktifkan α2AR pada membran presinaptik pada *posterior horn* dan membran postsinaptik neuron untuk menyebabkan hiperpolarisasi sel membran pada medulla spinalis. Pada saraf perifer, DEX menghambat aktivasi neuron nosiseptif melalui serabut saraf Aδ dan serabut saraf tipe-C dan pelepasan neurotransmitter nosiseptif: substans P. (34)

Pada penelitian ini, terdapat tiga faktor perancu yang dapat mempengaruhi agitasi pasca operasi yaitu nyeri pasca pembedahan, usia anak umur 2-5 tahun dan tingkat kecemasan anak. (21) Tatalaksana nyeri ditangani dengan pemberian Paracetamol 15mg/kg berat badan pada semua anak yang menjalani pembedahan. Mayoritas anak saat dipisahkan dari orang tua akan mengalami kecemasan namun kecemasan pra operasi akan tetap diukur dengan alat ukur *Modified Yale Preoperative Anxiety scale – short form (mYPAS-SF)* (Lampiran 7). (52) Skala mYPAS diatas 29 memiliki sensitivitas tinggi (78.8%) ntuk mengukur kecemasan praoperasi. (56) Penelitian oleh Moore et. al (3) menemukan bahwa anak berusia 2-5 tahun lebih rentan untuk mengalami agitasi pasca operasi, sehingga akan menjadi kriteria inklusi pada penelitian ini.

Dexmedetomidine menunjukkan efek sedatif hingga hipnotik tergantung dengan dosis yang digunakan tanpa mengganggu fungsi memori dan kognitif pasca paparan. Efek sedasi DEX bersifat endogen sehingga menyerupai kondisi tidur fisiologis tanpa timbulnya agitasi pasca pembiusan. Dengan demikian, penelitian

ini untuk bertujuan untuk mengevaluasi dan membandingkan insiden agitasi antara total anestesi intravena Dexmedotomidine dengan anestesi inhalasi Sevoflurane.

3.3 Hipotesis Penelitian

Kejadian agitasi pasca anestesi dengan anestesi intravena Dexmedetomidine lebih rendah dibandingkan dengan anestesi inhalasi Sevoflurane pada operasi bibir sumbing dan celah langit.

BAB 4

METODE PENELITIAN

4.1 Rancangan Penelitian

4.1.1 Jenis dan rancangan penelitian

Penelitian ini merupakan bagian dari uji experimental klinis dengan menggunakan desain post-test *Randomized Controlled Trial* (RCT) dengan metode *Single Blind*. Pada penelitian ini terdapat 2 kelompok, dengan masing – masing dilakukan 1 kali pengamatan, yaitu pasca ekstubasi dan setiap interval 15 menit di ruang pemulihan. Sebelum membagi menjadi 2 kelompok, tiap kelompok akan dilakukan pengkodean secara random dengan komputer sebelum dibagi menjadi kelompok kontrol dan eksperimental .

Skema Rancangan Penelitian:

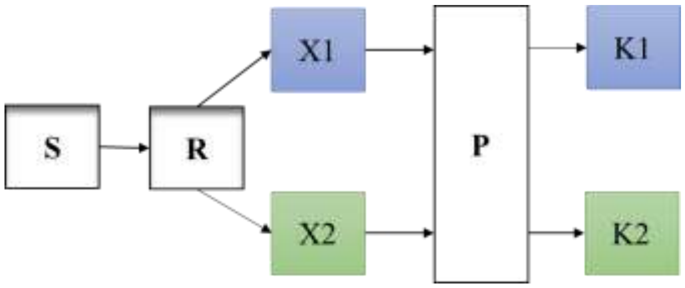

Keterangan:

S : Sampel Penelitian

R : Randomisasi

X1 : Kelompok subjek penelitian yang diberikan DEX

X2 : Kelompok kontrol penelitian Sevoflurane

P : Operasi bibir sumbing atau celah langit dengan pembiusan umum

K1 : Observasi skala agitasi dan komplikasi pasca operasi pada kelompok DEX

K2 : Observasi skala agitasi dan komplikasi pasca operasi pada kelompok Sevoflurane

4.2 Populasi, Besar Sampel dan Teknik Pengambilan Sampel

4.2.1 Populasi target

Populasi target adalah seluruh pasien anak usia 2 tahun – 5 tahun di Siloam Hospital Lippo Village.

4.2.2 Populasi terjangkau

Populasi terjangkau adalah seluruh pasien anak usia 2 tahun – 5 tahun yang menjalani pembedahan elektif bibir sumbing atau Siloam Hospital Lippo Village.

4.2.3 Sampel penelitian

Sampel penelitian adalah populasi terjangkau yang memenuhi kriteria inklusi dan tidak memenuhi kriteria ekslusi.

4.2.4 Kriteria inklusi

1. Pasien anak usia 2 – 5 tahun
2. Pasien dengan berat badan 5kg-25kg
3. Pasien dengan keadaan status fisik ASA 1-2
4. Setuju ikut dalam penelitian

4.2.5 Kriteria eksklusi

1. Pasien dengan kelainan anatomi, fungsi dan irama jantung
2. Pasien dengan kelainan kongenital bawaan atau sindrom : *Pierre Robin sequence*, Van der Woude sindrom, sindrom Stickler, *Craniofacial dysmorphia*, trisomy 21, trisomy 13, sindrom Velocardiofacial, sindrom Treacher Collins, sindrom Goldenhar (*hemifacial microsomia*).
3. Pasien dengan riwayat epilepsi
4. Pasien dengan gangguan fungsi hati

4.2.6 Kriteria *drop-out:*

1. Pasien/Wali pasien yang ingin menarik diri sepenuhnya dari penelitian (penarikan *informed consent*)
2. Terjadi alergi sistemik, reaksi anafilaktik, dan henti jantung.
3. Perdarahan lebih dari 20% *estimated blood volume*
4. Terjadi penyulit intraoperatif seperti perdarahan banyak saat operasi atau mengalami kegawat daruratan pada periode perioperatif.
5. Dalam jangka waktu 24 jam pascaoperasi, pasien mengalami perburukan kondisi sehingga harus dipindahkan pada perawatan *High Care Unit* ataupun *Intensive Care Unit*

4.2.7 Perkiraan besar sampel

Penelitian ini melihat perbandingan kejadian agitasi antara kelompok Sevoflurane dan kelompok Dexmedetomidine. Penelitian ini adalah penelitian analitik komparatif kategorik tidak berpasangan sehingga rumus sampel menggunakan rumus:

$$n1 = n2 = \left(\frac{Z\alpha\sqrt{2PQ} + Z\beta\sqrt{P1Q1 + P2Q2}}{P1 - P2} \right)^2$$

Keterangan:

n = besar masing-masing kelompok sampel

α = simpangan rata-rata dari tabel distribusi Z untuk α = 0,05, maka Zα =1.96 β = power test, ditetapkan peneliti β= 0. 20; maka Zβ = 0.84

Nilai p1= Proporsi pasien dengan agitasi pada kelompok X1 (Dexmedetomidine) = 10%

Nilai p2= Proporsi pasien dengan agitasi pada kelompok X2 (Sevoflurane) = 60%

Penelitian pendahuluan digunakan penelitian Peng dan Zhang, 2015. (6) Didapatkan sampel penelitian 13 sampel untuk masing – masing kelompok, dengan mengingat kemungkinan *drop out* pasien sebanyak 20%, maka dilakukan pengambilan sampel sebanyak 16 pasien untuk masing – masing kelompok dengan total 32 pasien secara keseluruhan.

4.2.8 Cara pengambilan sampel

Pada penelitian ini metode *consecutive sampling* dengan kedatangan sampel tanpa dimanipulasi oleh peneliti, sesuai dengan kriteria inklusi dan ekslusi. Dengan metode konsekutif, penelitian ini akan mengambil semua subjek yang memenuhi kriteria hingga jumlah sampel terpenuhi.

4.2.9 Prosedur penelitian

1. Prosedur penelitian dilakukan setelah memperoleh surat persetujuan dari komite etik penelitan Fakultas Kedokteran Universitas Pelita Harapan dan komite etik penelitian Siloam Hospital Lippo Village.
2. Identifikasi subjek penelitian untuk menilai kelayakan.
3. Setelah orang tua atau wali pasien diberikan penjelasan tentang prosedur penelitian dan setuju untuk ikut serta dalam penelitian, orang tua atau wali pasien akan diminta untuk menandatangani persetujuan. Identitas dan data prabedah dicatat.
4. Pasien dilakukan randomisasi dengan menggunakan randomisasi sederhana, dari angka 1 hingga 10. Randomisasi dilakukan menggunakan aplikasi randomizer.org. Randomisasi dilakukan secara acak menggunakan urutan angka yang dihasilkan komputer dengan sebuah angka yang tertutup dalam amplop. Pasien atau wali pasien akan diminta untuk mengambil kartu yang tertutup

amplop tersebut oleh koordinator penelitian, setelah dilakukan penilaian pra-operatif.

5. Pasien menjalankan puasa sesuai protokol puasa. Makan padat atau susu formula terakhir dikonsumsi hingga 6 jam sebelum operasi; 4 jam untuk ASI dan 2 jam untuk air putih.
6. Pada saat pasien datang di ruang persiapan, dilakukan penilaian kecemasan dengan skala *Modified Yale Preoperative Anxiety scale - Short form* (mYPAS-SF) dan dilakukan proses *sign in*. Kemudian anak dibawa masuk kedalam kamar operasi tanpa premedikasi.
7. Di kamar operasi dilakukan pemasangan monitor saturasi, elektrokardiografi dan tekanan darah noninvasif.
8. Induksi dilakukan dengan anestesi inhalasi Sevoflurane 8% sampai pasien tertidur dan pemasangan kanul intravena dilakukan. Kemudian, pasien diberikan Fentanyl 2ug/kg dan Propofol 3mg/kg secara intravena dan dilakukan pemasangan pipa endotrakeal atau *Laryngeal mask airway*. Anestesi inhalasi kemudian dihentikan dan pasien dibiarkan bernapas spontan dengan aliran oksigen 100%, 4-5 Liter per menit. Cairan *Ringer Lactate* diberikan sebanyak 4ml/kg/jam pada setiap pasien.
8. Selama operasi, pemantauan hemodinamik (saturasi oksigen darah, tekanan darah dan elektrokardiografi) dan kesadaran pasien dipantau secara kontinu. Jika terjadi peningkatan kadar PSI diatas 50, maka pasien akan diberikan *Rescue Propofol* secara bolus intravena untuk menjaga kedalaman anestesi dalam rentang PSI 25-50.

9. Jika terjadi bradikardia, maka pasien akan diberikan obat emergensi berupa Atropine 0.02mg/kg secara bolus intravena. Kebutuhan total *Rescue Propofol* maupun kejadian bradikardia atau hipotensi akan dicatat dan dilaporkan pada penelitian ini.
10. Setelah operasi selesai, pasien akan dibangunkan dan dinilai waktu ekstubasi, dan skala agitasi. Pasien akan dipindahkan ruang pemulihan dimana pasien akan dipantau secara ketat untuk hemodinamik, kejadian desaturasi, laringospasme, dan skala agitasi yang diukur setiap 15 menit sampai pasien sadar secara sepenuhnya.
11. Pasca operasi, nyeri akan dijaga dengan target FLACC dibawah 4. Jika terjadi FLACC diatas 4, maka pasien akan diberikan *rescue* analgetik berupa Fentanyl 1ug/kg bolus intravena dan skala nyeri dipantau sampai tercapai skala FLACC dibawah 4.
12. Bila terjadi agitasi, pasien akan dijaga agar tidak terjadi insiden jatuh dan tingkat kesadaran dinilai sampai pasien sadar pulih dan aman untuk dipindahkan ke ruang rawat inap. Skala agitasi akan tetap diukur setiap 15 menit.

4.3 Variabel Penelitian dan Definisi Operasional

4.3.1 Variabel bebas

Variabel bebas adalah anestesi inhalasi Sevoflurane dan anestesi intravena Dexmedetomidine.

4.3.2 Variabel tergantung

Variabel tergantung adalah skala agitasi

4.3.3 Variabel perancu

Variabel perancu adalah tingkat kecemasan anak pra-operasi yang akan diukur dengan skala mYPAS.

4.3.4 Definisi operasional

Tabel 4.1 Definisi operasional

No	Variabel	Definisi Operasional	Dosis / Ukuran / Satuan	Skala
1	Anestesi Inhalasi Sevoflurane	Teknik anestesi umum menggunakan obat anestesi inhalasi Sevoflurane	-Inhalasi Sevoflurane menggunakan 1-1.5 MAC sehingga tercapai PSI 25-50	Nominal
2	Anestesi Intravena Dexmedeto midine	Teknik anestesi umum menggunakan obat anestesi intravena Dexmedetomidine	-Intravena Dexmedetomidine menggunakan *Loading dose* 1.5µg/kg bolus selama 10 menit via syringe pump diikuti oleh *maintanance dose* / 1.5 µg/kg/jam sehingga tercapai PSI 25-50	Nominal
2	Tingkat kecemasan	Skala yang digunakan untuk mengukur kecemasan anak pra-operasi dengan *Modified Yale Preoperative Anxiety scale- short form (mYPAS SF)*	Skor mYPAS dibawah 29 dianggap tidak cemas; sedangkan skor diatas 29 dianggap sebagai cemas	Ordinal
4	*Rescue Propofol*	Obat anestesi intravena yang diberikan saat nilai PSI diatas 50 pada fase intra-operatif	10mg/ml	Numerik
5	Agitasi pasca anestesi	Kejadian agitasi yang dinilai setelah anak dibangunkan dari pembiusan dan dinilai dengan Cravero Scale.	Skala agitasi yang dinilai mulai dari ekstubasi dan dilanjutkan setiap interval 15 menit di ruang pemulihan sampai pasien pulih sadar	Numerik

4.4 Materi Penelitian

Penilaian agitasi pasca operasi bibir sumbing dan/atau celah langit.

4.5 Instrumen Penelitian

4.5.1 SEDLine™ monitor, Masimo Corporation, Irvine, CA, USA

Sedline monitor adalah alat untuk mengukur *Patient State Index* (PSI) yang mengukur elektroensefalografi dan tingkat kesadaran selama pembedahan berjalan. Gelombang EEG dikonversikan menjadi nilai 25-50 yang di definisikan sebagai kondisi anestesia.

4.5.2 *Modified Yale Pre-operative Anxiety Scale – Short Form* (mYPAS-SF)

mYPAS-SF adalah alat pengukur kecemasan pada anak umur 2 tahun hingga 12 tahun. Nilai dibawah 29 dianggap sebagai tidak cemas, sedangkan nilai 29 dan diatasnya dianggap sebagai anak cemas.

4.5.3 *Syringe pump*

Alat medis yang digunakan untuk mengatur proses penyuntikan atau injeksi cairan maupun dosis obat ke dalam tubuh pasien dalam kuantitas waktu tertentu.

4.5.4 Waktu ekstubasi

Waktu esktubasi akan dihitung dimulai saat infus Dexmedetomidine dihentikan atau inhalasi Sevoflurane dihentikan sampai pada pasien dilakukan ekstubasi pipa endotrakeal atau eksersi *Laryngeal Mask*.

4.5.5 Waktu pulih sadar

Waktu pulih sadar akan dihitung dimulai saat infus Dexmedetomidine dihentikan atau inhalasi Sevoflurane dihentikan sampai pada saat pasien sadar

sepenuhnya (mampu mengenali pengasuh atau orang tua) dan memberikan respon yang sesuai, seperti sebelum menjalani pembiusan.

4.5.5 Skala agitasi

Skala agitasi akan dinilai menggunakan skala Cravero. Penilaian pertama dilakukan di kamar operasi setelah pasien dilakukan ekstubasi atau eksersi LMA; penilaian selanjutnya dilakukan di ruang pemulihan dan dinilai setiap interval 15 menit sampai pasien pulih sadar secara sepenuhnya.

4.6 Lokasi dan Waktu Penelitian

Penelitian dilaksanakan di Siloam Hospital Lippo Village setelah mendapat persetujuan dari komite etik penelitian Siloam Hospital Lippo Village dan komite etik penelitian Fakultas Kedokteran Universitas Pelita Harapan yang dilaksanakan pada 3 Mei 2024 hingga 31 Desember 2024.

4.7 Kerangka Operasional Penelitian

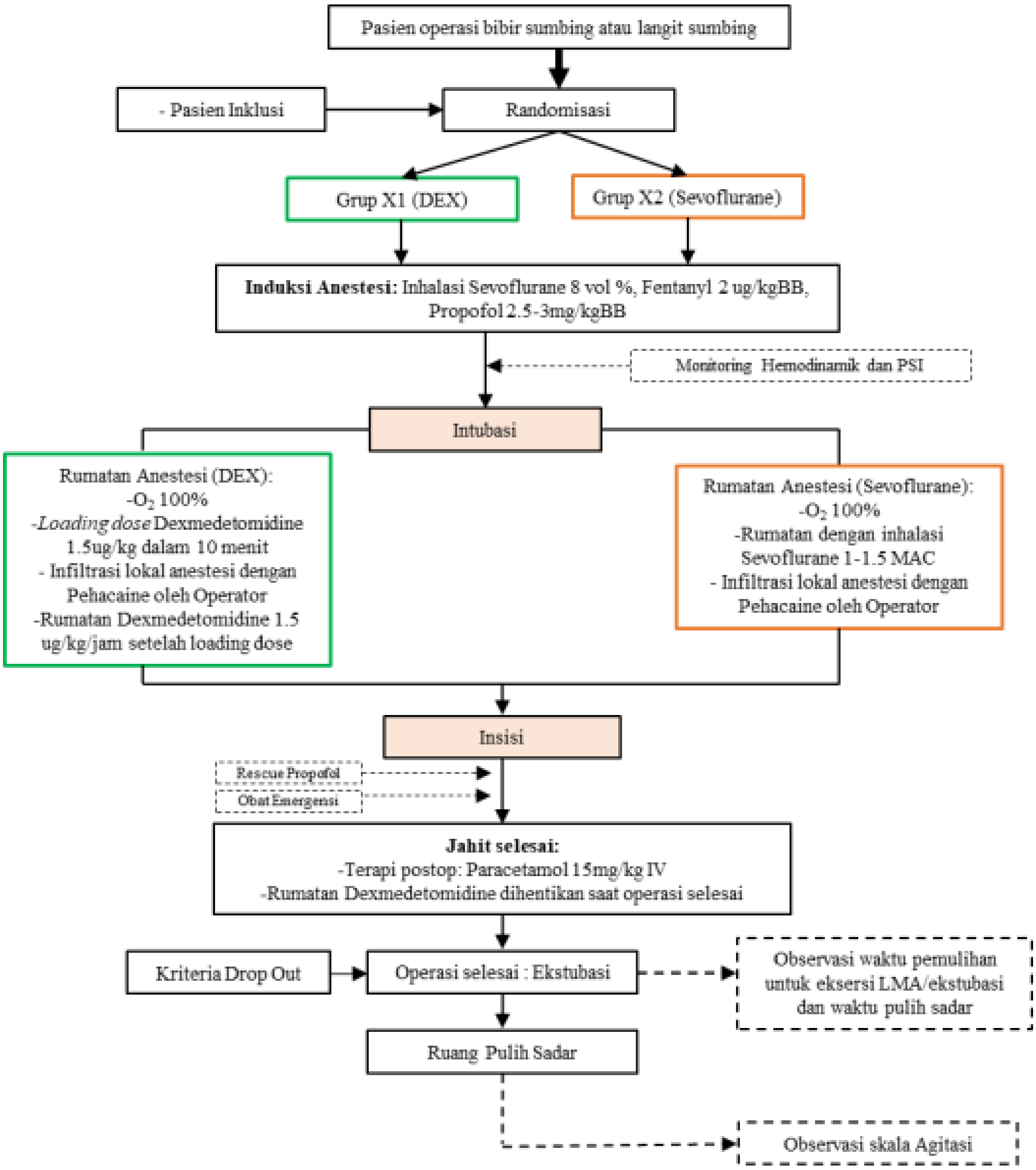

Gambar 4.1 Kerangka operasional penelitian

4.8 Pengelolaan dan Analisis Data

Data hasil penelitian disajikan secara deskriptif dalam bentuk tabel dan grafik untuk menggambarkan grup kelompok Dexmedetomidine dibandingkan grup Sevoflurane. Beda antara dua kelompok dilakukan uji beda dengan *Chi-square*.

4.9 Risiko Penelitian

Pemberian obat anestesi Dexmedetomidine dapat menimbulkan efek samping berupa bradikardia dan hipotensi. Bila terjadi bradikardia atau hipotensi, maka akan diberikan Atropine 0.02mg/kg atau Epinephrine 0.01mg/kg secara bolus intravena.

4.10 Etika Penelitian

Penelitian ini akan dilakukan setelah mendapatkan surat keterangan lolos kaji etik dari komite etik penelitian Fakultas Kedokteran Universitas Pelita Harapan dan komite etik penelitian Siloam Hospital Lippo Village. Orang tua atau wali pasien yang memenuhi kriteria penerimaan akan diberikan informasi penelitian dengan jelas dan diminta untuk menandatangani lembar *informed consent* sebagai syarat setuju untuk ikut berpartisipasi dalam penelitian. Informasi akan diberikan kepada orang tua atau wali pasien meliputi tujuan dan manfaat penelitian, cara dan prosedur penelitian, jumlah subjek penelitian, waktu penelitian, risiko dan efek samping tindakan yang akan dilakukan, kompensasi atau penanganan jika terjadi efek samping atau risiko yang tidak diinginkan, penanganan kejadian yang tidak diinginkan, penjagaan informasi rahasia subjek, biaya yang ditanggung subjek, insentif bagi subjek, nama dan alamat peneliti yang harus dihubungi jika terjadi efek samping atau subjek ingin bertanya. Pasien dan/atau keluarga berhak menerima, menolak, atau membatalkan untuk berpartisipasi dalam penelitian dengan sukarela tanpa paksaan dari pihak manapun. Pasien dan/atau keluarga didampingi wali dalam mengisi lembar *informed consent* penelitian sebagai bentuk pernyataan menerima atau menolak berpartisipasi dalam penelitian. Pasien yang

menolak atau subjek yang membatalkan/keluar dari penelitian saat penelitian masih berjalan akan tetap mendapatkan pelayanan sesuai standar yang berlaku.

DAFTAR PUSTAKA

1. Voepel-Lewis T, Malviya S, Tait AR. A Prospective Cohort Study of Emergence Agitation in the Pediatric Postanesthesia Care Unit. Anesth Analg [Internet]. 2003;96(6). Available from: https://journals.lww.com/anesthesia-analgesia/fulltext/2003/06000/a_prospective_cohort_study_of_emergence_agitation.16.aspx

2. Liu K, Liu C, Ulualp SO. Prevalence of Emergence Delirium in Children Undergoing Tonsillectomy and Adenoidectomy. Minervini G, editor. Anesthesiol Res Pract [Internet]. 2022;2022:1465999. Available from: https://doi.org/10.1155/2022/1465999

3. Moore AD, Anghelescu DL. Emergence Delirium in Pediatric Anesthesia. Paediatr Drugs. 2017 Feb;19(1):11–20.

4. Menser C, Smith H. Emergence Agitation and Delirium: Considerations for Epidemiology and Routine Monitoring in Pediatric Patients. Local Reg Anesth. 2020;13:73–83.

5. Reduque LL, Verghese ST. Paediatric emergence delirium. Contin Educ Anaesth Crit Care Pain [Internet]. 2013 Apr 1;13(2):39–41. Available from: https://doi.org/10.1093/bjaceaccp/mks051

6. Peng W, Zhang T. Dexmedetomidine decreases the emergence agitation in infant patients undergoing cleft palate repair surgery after general anesthesia. BMC Anesthesiol [Internet]. 2015;15(1):145. Available from: https://doi.org/10.1186/s12871-015-0124-7

7. Cravero J, Surgenor S, Whalen K. Emergence agitation in paediatric patients after sevoflurane anaesthesia and no surgery: a comparison with halothane. Paediatr Anaesth. 2000;10(4):419–24.

8. Andriyanto L, Utariani A, Hanindito E, Santoso KH, Puspita EA. Incidence of Emergence Agitation in Pediatric Patient after general anesthesia. Folia Medica Indones. 2019;55(March):25–9.

9. Desalu I, Adeyemo W, Akintimoye M, Adepoju A. Airway and respiratory complications in children undergoing cleft lip and palate repair. Ghana Med J. 2010 Mar;44(1):16–20.

10. Zhang J, Yin J, Li Y, Zhang Y, Bai Y, Yang H. Effect of dexmedetomidine on preventing perioperative respiratory adverse events in children: A systematic review and meta-analysis of randomized controlled trials. Exp Ther Med. 2023 Jun;25(6):286.

11. Li L, Zhang Z, Yao Z, Wang H, Wang H, An H, et al. The impact of laryngeal mask versus other airways on perioperative respiratory adverse events in

children: A systematic review and meta-analysis of randomized controlled trials. Int J Surg. 2019 Apr;64:40–8.

12. Kulkarni KR, Patil MR, Shirke AM, Jadhav SB. Perioperative respiratory complications in cleft lip and palate repairs: An audit of 1000 cases under “Smile Train Project”. Indian J Anaesth. 2013 Nov;57(6):562–8.

13. Liu D, Pan L, Gao Y, Liu J, Li F, Li X, et al. Efficaciousness of dexmedetomidine in children undergoing cleft lip and palate repair: a systematic review and meta-analysis. BMJ Open. 2021 Aug;11(8):e046798.

14. Surana P, Parikh DA, Patkar GA, Tendolkar BA. A prospective randomized controlled double-blind trial to assess the effects of dexmedetomidine during cleft palate surgery. Korean J Anesthesiol [Internet]. 2017;70(6):633–41. Available from: http://europepmc.org/abstract/MED/29225747

15. Zhang X, Bai Y, Shi M, Ming S, Jin X, Xie Y. Effect of different administration and dosage of dexmedetomidine in the reduction of emergence agitation in children: a meta-analysis of randomized controlled trials with sequential trial analysis. Transl Pediatr [Internet]. 2021;10(4):929–57. Available from: http://europepmc.org/abstract/MED/34012842

16. Freriksen JJM, van der Zanden TM, Holsappel IGA, Molenbuur B, de Wildt SN. Best Evidence-Based Dosing Recommendations for Dexmedetomidine for Premedication and Procedural Sedation in Pediatrics: Outcome of a Risk-Benefit Analysis By the Dutch Pediatric Formulary. Paediatr Drugs. 2022 May;24(3):247–57.

17. Weerink MAS, Struys MMRF, Hannivoort LN, Barends CRM, Absalom AR, Colin P. Clinical Pharmacokinetics and Pharmacodynamics of Dexmedetomidine. Clin Pharmacokinet [Internet]. 2017;56(8):893–913. Available from: https://doi.org/10.1007/s40262-017-0507-7

18. Garcia PS, Kolesky SE, Jenkins A. General anesthetic actions on GABA(A) receptors. Curr Neuropharmacol. 2010 Mar;8(1):2–9.

19. Sakai EM, Connolly LA, Klauck JA. Inhalation anesthesiology and volatile liquid anesthetics: focus on isoflurane, desflurane, and sevoflurane. Pharmacotherapy. 2005 Dec;25(12):1773–88.

20. Torri G. Inhalation anesthetics: a review. Minerva Anestesiol. 2010 Mar;76(3):215–28.

21. Lee S-J, Sung T-Y. Emergence agitation: current knowledge and unresolved questions. Korean J Anesthesiol. 2020 Dec;73(6):471–85.

22. Kim JH. Mechanism of emergence agitation induced by sevoflurane anesthesia. Korean J Anesthesiol. 2011 Feb;60(2):73–4.

23. Mason KP. Paediatric emergence delirium: a comprehensive review and interpretation of the literature. Br J Anaesth. 2017 Mar;118(3):335–43.

24. Doerrfuss JI, Kramer S, Tafelski S, Spies CD, Wernecke K-D, Nachtigall I. Frequency, predictive factors and therapy of emergence delirium: data from a large observational clinical trial in a broad spectrum of postoperative pediatric patients. Minerva Anestesiol. 2019 Jun;85(6):617–24.

25. Kayyal TA, Wolfswinkel EM, Weathers WM, Capehart SJ, Monson LA, Buchanan EP, et al. Treatment effects of dexmedetomidine and ketamine on postoperative analgesia after cleft palate repair. Craniomaxillofac Trauma Reconstr. 2014 Jun;7(2):131–8.

26. Nair S, Wolf A. Emergence delirium after paediatric anaesthesia: new strategies in avoidance and treatment. BJA Educ. 2018 Jan;18(1):30–3.

27. Mapelli J, Gandolfi D, Giuliani E, Casali S, Congi L, Barbieri A, et al. The effects of the general anesthetic sevoflurane on neurotransmission: an experimental and computational study. Sci Rep [Internet]. 2021;11(1):4335. Available from: https://doi.org/10.1038/s41598-021-83714-y

28. Massimini M, Ferrarelli F, Huber R, Esser SK, Singh H, Tononi G. Breakdown of cortical effective connectivity during sleep. Science. 2005 Sep;309(5744):2228–32.

29. Yang L, Ton H, Zhao R, Geron E, Li M, Dong Y, et al. Sevoflurane induces neuronal activation and behavioral hyperactivity in young mice. Sci Rep [Internet]. 2020;10(1):11226. Available from: https://doi.org/10.1038/s41598-020-66959-x

30. Goa KL, Noble S, Spencer CM. Sevoflurane in paediatric anaesthesia: a review. Paediatr Drugs. 1999;1(2):127–53.

31. Flacker JM, Lipsitz LA. Neural mechanisms of delirium: current hypotheses and evolving concepts. J Gerontol A Biol Sci Med Sci. 1999 Jun;54(6):B239-46.

32. Samuels ER, Szabadi E. Functional neuroanatomy of the noradrenergic locus coeruleus: its roles in the regulation of arousal and autonomic function part I: principles of functional organisation. Curr Neuropharmacol. 2008 Sep;6(3):235–53.

33. Toyoda Y, Zhu A, Kong F, Shan S, Zhao J, Wang N, et al. Structural basis of α1A-adrenergic receptor activation and recognition by an extracellular nanobody. Nat Commun. 2023;14(1):1–13.

34. Liu X, Li Y, Kang L, Wang Q. Recent Advances in the Clinical Value and Potential of Dexmedetomidine. J Inflamm Res. 2021;14:7507–27.

35. Coeckelenbergh S, Doria S, Patricio D, Perrin L, Engelman E, Rodriguez A, et al. Effect of dexmedetomidine on Nociception Level Index-guided

remifentanil antinociception: A randomised controlled trial. Eur J Anaesthesiol. 2021 May;38(5):524–33.

36. Sottas CE, Anderson BJ. Dexmedetomidine: the new all-in-one drug in paediatric anaesthesia? Curr Opin Anaesthesiol. 2017 Aug;30(4):441–51.

37. Bailey CR. Dexmedetomidine in children – when should we be using it? Anaesthesia [Internet]. 2021;76(3):309–11. Available from: https://associationofanaesthetists-publications.onlinelibrary.wiley.com/doi/abs/10.1111/anae.15169

38. Gertler R, Brown HC, Mitchell DH, Silvius EN. Dexmedetomidine: a novel sedative-analgesic agent. Proc (Bayl Univ Med Cent). 2001 Jan;14(1):13–21.

39. Di M, Huang C, Chen F, Zeng R, Yu C, Shangguan W, et al. [Effect of single-dose dexmedetomidine on recovery profiles after sevoflurane anesthesia with spontaneous respiration in pediatric patients undergoing cleft lip and palate repair]. Zhonghua Yi Xue Za Zhi [Internet]. 2014;94(19):1466–9. Available from: http://europepmc.org/abstract/MED/25143165

40. Mahmoud M, Barbi E, Mason KP. Dexmedetomidine: What's New for Pediatrics? A Narrative Review. J Clin Med. 2020 Aug;9(9).

41. Naaz S, Ozair E. Dexmedetomidine in current anaesthesia practice- a review. J Clin Diagn Res. 2014 Oct;8(10):GE01-4.

42. Su F, Hammer GB. Dexmedetomidine: pediatric pharmacology, clinical uses and safety. Expert Opin Drug Saf. 2011 Jan;10(1):55–66.

43. Denning S, Ng E, Wong Riff KWY. Anaesthesia for cleft lip and palate surgery. BJA Educ. 2021 Oct;21(10):384–9.

44. Machotta A. [Anesthetic management of pediatric cleft lip and cleft palate repair]. Anaesthesist. 2005 May;54(5):455–66.

45. Vyas T, Gupta P, Kumar S, Gupta R, Gupta T, Singh HP. Cleft of lip and palate: A review. J Fam Med Prim care. 2020 Jun;9(6):2621–5.

46. Fontanals M, Merritt G, Sierra P, Echaniz G. Anesthetic Considerations and Complications of Cleft Palate Repairs. What's New? Curr Anesthesiol Rep [Internet]. 2021;11(3):257–64. Available from: https://doi.org/10.1007/s40140-021-00460-7

47. Drover D, Ortega HR (Rick). Patient state index. Best Pract Res Clin Anaesthesiol [Internet]. 2006;20(1):121–8. Available from: https://www.sciencedirect.com/science/article/pii/S152168960500056X

48. Ricci Z, Robino C, Rufini P, Cumbo S, Cavallini S, Gobbi L, et al. Monitoring anesthesia depth with patient state index during pediatric surgery. Pediatr Anesth [Internet]. 2023 Oct 1;33(10):855–61. Available

from: https://doi.org/10.1111/pan.14711

49. Sciusco A, Standing JF, Sheng Y, Raimondo P, Cinnella G, Dambrosio M. Effect of age on the performance of bispectral and entropy indices during sevoflurane pediatric anesthesia: a pharmacometric study. Pediatr Anesth [Internet]. 2017 Apr 1;27(4):399–408. Available from: https://doi.org/10.1111/pan.13086

50. Jang Y-E, Kim E-H, Lee J-H, Kim J-T, Kim H-S. Usefulness of bispectral index and patient state index during sevoflurane anesthesia in children: A prospective observational study. Medicine (Baltimore). 2022 Jul;101(30):e29925.

51. Soehle M, Ellerkmann RK, Grube M, Kuech M, Wirz S, Hoeft A, et al. Comparison between Bispectral Index and Patient State Index as Measures of the Electroencephalographic Effects of Sevoflurane. Anesthesiology [Internet]. 2008 Nov 1;109(5):799–805. Available from: https://doi.org/10.1097/ALN.0b013e3181895fd0

52. Jenkins BN, Fortier MA, Kaplan SH, Mayes LC, Kain ZN. Development of a short version of the modified Yale Preoperative Anxiety Scale. Anesth Analg. 2014 Sep;119(3):643–50.

53. Russell PSS, Mammen PM, Shankar SR, Viswanathan SA, Rebekah G, Russell S, et al. Pediatric Anesthesia Emergence Delirium Scale: A diagnostic meta-analysis. World J Clin Pediatr. 2022 Mar;11(2):196–205.

54. Kong H, Li M, Deng C-M, Wu Y-J, He S-T, Mu D-L. A comprehensive overview of clinical research on dexmedetomidine in the past 2 decades: A bibliometric analysis. Front Pharmacol. 2023;14:1043956.

55. Mahmoud M, Mason KP. Dexmedetomidine: review, update, and future considerations of paediatric perioperative and periprocedural applications and limitations. Br J Anaesth [Internet]. 2015 Aug 1;115(2):171–82. Available from: https://doi.org/10.1093/bja/aev226

56. Prayunanto E, Widyastuti Y, Sari D. The association of modified Yale perioperative anxiety scale and pediatric anesthesia behavior on postoperative emergence delirium in children: A prospective cohort study. Bali J Anesthesiol [Internet]. 2023;7(2). Available from: https://journals.lww.com/bjoa/fulltext/2023/07020/the_association_of_modified_yale_perioperative.6.aspx

Lampiran 1. Lembar Penjelasan Penelitian

LEMBAR PENJELASAN KEPADA CALON SUBJEK

Saya, dr. Corry Quando Yahya, Sp.An , Tim Peneliti yang diketuai oleh Dr. Kohar Hari Santoso, dr., SpAn-TI., Subsp.An.Ped.(K)., Subsp TI(K), dari Departemen Anestesiologi dan Terapi Intensif, FK Unair dan Universitas Pelita Harapan akan melakukan penelitian dengan judul Perbandingan kejadian agitasi anestesi intravena Dexmedetomidine dengan inhalasi Sevoflurane pada operasi bibir sumbing dan celah langit.

Saya akan memberikan informasi kepada (Bapak/Ibu/Saudara) mengenai penelitian ini dan mengundang putra/putri (Bapak/Ibu/Saudara) untuk menjadi bagian dari penelitian ini.

Bapak/Ibu/Saudara dapat berpartisipasi dalam penelitian ini dengan cara menandatangani formulir ini. Jika Bapak/Ibu/Saudara setuju untuk berpartisipasi dalam penelitian ini, Bapak/Ibu/Saudara kapan saja dapat secara bebas mundur dari penelitian ini. Jika Bapak/Ibu/Saudara menolak untuk berpartisipasi atau mundur dari penelitian ini, keputusan tersebut tidak akan mempengaruhi hubungan Bapak/Ibu/Saudara dengan saya dan tidak akan berdampak pada pelayanan yang berlaku di rumah sakit ini.

Jika Bapak/Ibu/Saudara tidak mengerti tiap pernyataan dalam formulir ini, Bapak/Ibu/Saudara dapat menanyakannya kepada saya.

Tujuan Penelitian

Pada penelitian ini, putra/putri (Bapak/Ibu/Saudara) akan diberikan obat bius bernama Dexmedetomidine lewat pembuluh darah vena. Obat ini bertujuan untuk memberikan efek tertidur pada anak (Bapak/Ibu/Saudara) selama prosedur operasi berlangsung. Efek samping dari pemberian obat Dexmedetomidine sebagai obat bius berupa penurunan tekanan darah dan penurunan detak jantung. Jika terdapat efek samping tersebut, maka anak akan diberikan obat untuk meningkatkan tekanan darah dan detak jantung. Dalam penelitian ini, waktu untuk pulih dari pembiusan

dan efek samping seperti agitasi (gelisah) dan penurunan kadar oksigen dalam darah pasca pembiusan akan dicatat dan dibandingkan dengan pemberian obat bius inhalasi (Sevoflurane). Melalui penelitian ini diharapkan putra/putri (Bapak/Ibu/Saudara) akan mendapatkan kenyamanan dan keamanan saat pulih dari pembiusan sehingga dapat mempercepat penyembuhan anak pasca bedah.

Partisipasi dalam penelitian

Secara keseluruhan, penelitian akan berjalan hingga target jumlah subjek yang ditentukan telah terpenuhi (perkiraan 1 tahun). Apabila Bapak/Ibu/ Saudara memutuskan ikut dalam penelitian ini, putra/putri Bapak/Ibu/Saudara akan mendapatkan obat bius Dexmedetomidine selama operasi berlangsung.

Alasan memilih Bapak/Ibu/Saudara

Putra/putri Bapak/Ibu/Saudara dipilih sesuai dengan kriteria penelitian ini yaitu pasien anak usia 2 – 5 tahun dengan penilaian status fisik ASA 1 dan 2 (sakit ringan), memiliki toleransi pemberian cairan enteral (peroral) yang baik, tidak memiliki kelainan sistem hormon dan akan menjalani pembedahan bibir sumbing atau langit sumbing dengan anestesia umum.

Prosedur Penelitian

1. Identifikasi subjek penelitian untuk menilai kelayakan.
2. Setelah orang tua atau wali pasien diberikan penjelasan tentang prosedur penelitian dan setuju untuk ikut serta dalam penelitian, orang tua atau wali pasien akan diminta untuk menandatangani persetujuan. Identitas dan data prabedah dicatat.
3. Pasien akan dikelompokkan menjadi 2 kelompok secara acak, yaitu kelompok X1 yang akan menerima obat bius yaitu Dexmedetomidine secara intravena dan kelompok X2 yang akan mendapatkan obat bius berupa anestesi inhalasi, Sevoflurane. Obat anestesi Dexmedetomidine atau Sevoflurane adalah obat yang digunakan sebagai pemeliharaan anestesi selama proses pembedahan berlangsung sampai selesai.

4. Pasien menjalankan puasa selama protokol puasa. Makan padat terakhir dikonsumsi hingga 6 jam sebelum operasi, ASI sampai dengan 4 jam pra-operasi.
5. Pada saat pasien datang di ruang persiapan, dilakukan proses penerimaan pasien dan anak akan dibawa masuk kedalam ruang operasi untuk proses induksi anestesi.
6. Di kamar operasi dilakukan pemasangan monitor saturasi, elektrokardiografi dan tekanan darah nonivasif. Sebelum pembiusan, dilakukan pencatatan tekanan darah dan laju nadi pasien. Induksi dilakukan dengan standar pembiusan sesuai kebutuhan anak. Pemeliharaan anestesi dilakukan dengan Sevoflurane atau Dexmedetomidine sesuai dengan kelompok anak. Setelah operasi selesai, waktu pemulihan dan kejadian penurunan kadar oksigen darah atau kejadian gelisah akan dinilai pada dua kelompok.

Risiko

Efek samping dan tatalaksana dari pembiusan berupa penurunan tekanan darah dan penurunan detak jantung. Dari panduan terbaru untuk penanganan tersebut adalah pemberian cairan infus dan obat untuk menaikkan tekanan darah dan denyut nadi secara intravena. Obat dan cairan infus tersebut selalu disediakan pada setiap operasi.

Manfaat

Penelitian ini menjadi landasan awal untuk penggunaan Dexmedetomidine sebagai obat pemeliharaan anestesi anak yang menjalani operasi bibir sumbing dan celah langit.

Kompensasi

Tidak ada kompensasi atau imbalan dari partisipasi penelitian ini.

Pembiayaan

Penelitian ini dibiayai sepenuhnya oleh pihak peneliti.

Kerahasiaan

Semua data yang dikumpulkan dalam penelitian ini akan dijaga kerahasiaannya**.** Identitas setiap responden akan kami tampilkan dalam bentukinisial dan hanya bisa diakses oleh tim peneliti dan komite etik untuk verifikasi.Presentasi hasil penelitian dalam pertemuan ilmiah / konferensi dan publikasi dalam jurnal ilmiah tidak akan mencantumkan nama Bapak/Ibu/Saudara.

Kewajiban subyek penelitian

Sebagai subyek penelitian, Bapak/Ibu/Saudara berkewajiban mengikuti aturan atau petunjuk penelitian seperti yang tertulis di atas. Bila ada yang belum jelas, Bapak/Ibu/Saudara bisa bertanya lebih lanjut kepada tim peneliti.

Hak untuk menolak dan mengundurkan diri

Bapak/Ibu/Saudara tidak harus berpartisipasi dalam penelitian ini bila tidak menghendakinya. Bapak/Ibu/Saudara harus paham bahwa walaupun Bapak/Ibu/Saudara menyetujui untuk berpartisipasi, Bapak/Ibu/Saudara

berhak untuk mundur dari penelitian ini. Jika Bapak/Ibu/Saudara menolak untuk berpartisipasi atau mundur dari penelitian ini, keputusan tersebut tidak akan mempengaruhi hubungan Bapak/Ibu/Saudara dengan saya dan tidak akan berdampak pada standar pelayanan yang berlaku di rumah sakit ini. Saya akan memberikan kesempatan pada Bapak/Ibu/Saudara pada akhirpenjelasan ini untuk dapat mempertimbangkan keputusan yang akan diambil.

Akses pasca penelitian (*Post-trial access*)

Tidak ada

Informasi Tambahan

Bapak/ ibu/ saudara diberi kesempatan untuk menanyakan semua hal yang belum jelas sehubungan dengan penelitian ini. Bila sewaktu-waktu membutuhkan penjelasan lebih lanjut, Bapak/ ibu/ saudara dapat menghubungi **dr. Corry Quando Yahya, Sp.An** pada no. HP 081383190900, **Dr. Kohar Hari Santoso, dr., SpAn-TI.,Subsp.An.Ped.(K).,Subsp TI(K) dan Lucky Andriyanto, dr., SpAn-TI., Subsp.An.Ped.(K)., Subsp TI (K),** di **Departemen Anestersiologi dan Terapi Intensif FK-UNAIR** dan **Fakultas Kedokteran Universitas Pelita Harapan**.

Lampiran 2. Lembar Persetujuan Partisipasi Penelitian

LEMBAR PERSETUJUAN KEIKUSERTAAN DALAM PENELITIAN

Semua penjelasan tersebut telah disampaikan kepada saya dan semua pertanyaan saya telah dijawab oleh **tim peneliti.** Saya mengerti bahwa bila memerlukan penjelasan, saya dapat menanyakan kepada **dr. Corry Quando Yahya, Sp.An.**

**Sertifikat Persetujuan (*Consent*)**	
Saya telah membaca semua penjelasan tentang penelitian ini. Saya telah diberikan kesempatan untuk bertanya dan semua pertanyaan saya telah dijawab dengan jelas. Saya bersedia untuk berpartisipasi pada studi penelitian ini dengan sukarela.<br><br>Nama subjek/wali	Saya mengkonfirmasi bahwa peserta telah diberikan kesempatan untuk bertanya mengenai penelitian ini, dan semua pertanyaan telah dijawab dengan benar. Saya mengkonfirmasi bahwa persetujuan telah diberikan dengan sukarela.
	Nama peneliti/peminta persetujuan
Tanda tangan wali peserta penelitian<br><br>Tanggal____________________<br><br>hari/bulan/tahun	Tanda tangan peneliti/peminta persetujuan<br><br>Tanggal____________________<br><br>hari/bulan/tahun

Informasi Peneliti

Peneliti Utama : **dr. Corry Quando Yahya, Sp.An-TI**
Jalan Palem Putri Raya No. 17A, Palem Semi, Tangerang 15810
HP: 081383190900
Email: corry.spa@gmail.com

Peneliti : **Dr. Kohar Hari Santoso, dr., SpAn-TI.,Subsp.An.Ped.(K).,Subsp TI(K)**
Lucky Andriyanto, dr., SpAn-TI., Subsp.An.Ped.(K)., Subsp TI (K)
Gedung Anestesiologi dan Terapi Intensif, FK UNAIR. Jl. Prof.Dr. Moestopo No 6-8, Kota Surabaya 60286

LEMBAR PERSETUJUAN

Untuk subjek penelitian **(…..)** yang ikut dalam penelitian: Perbandingan total anestesi intravena Dexmedetomidine dengan inhalasi Sevoflurane pada operasi bibir sumbing dan celah langit**.** Saya telah membaca dan mengerti informasi yang tercantum pada lembar informasi dan telah diberi kesempatan untuk mendiskusikan dan menanyakan hal tersebut. Saya setuju untukmengijinkan anak saya mendapatkan **tindakan sesuai protokol penelitian**. Saya mengerti bahwa saya dapat menolak untuk ikut dalam penelitian. Saya sadar bahwasaya dapat mengundurkan diri dari penelitian ini kapan saja saya mau. Saya mengerti bahwa apabula saya tidak mengikuti penelitian ini, anak saya tetap akan menerima perawatan medis yang semestinya **kondisi pasien**.

Saya, sebagai **ORANG TUA/WALI** dari ....

SETUJU untuk berpartisipasi dalam penelitian ini.

Tanggal : ……………………….............……......

Tanda tangan Orang Tua/Wali : ……………………………………….

Nama Orang Tua/Wali : **……………………………………...…**

Tanda tangan Saksi : ……………………………………..…

Nama Saksi : ……………………………….....……

Lampiran 3. Lembar Persetujuan untuk Publikasi

PERSETUJUAN PUBLIKASI

Saya memberikan persetujuan saya untuk materi tentang saya / pasien untuk muncul di Anestesi Anak.

Saya mengonfirmasi bahwa saya: (silakan centang untuk mengkonfirmasi)

- ○ telah melihat foto, gambar, teks atau materi lain tentang saya / pasien
- ○ telah membaca artikel yang akan diserahkan ke jurnal
- ○ Saya secara hukum berhak untuk memberikan persetujuan ini.

Saya memahami hal-hal berikut:

(1) Materi akan dipublikasikan tanpa nama saya / pasien terlampir, namun saya memahami bahwa anonimitas lengkap tidak dapat dijamin. Ada kemungkinan bahwa seseorang di suatu tempat - misalnya, seseorang yang merawat saya / pasien atau kerabat - dapat mengenali saya / pasien.

(2) Materi dapat menunjukkan atau menyertakan rincian kondisi medis atau cedera saya / pasien dan prognosis, perawatan atau operasi apa pun yang saya miliki / pasien telah, miliki atau mungkin miliki di masa depan.

(3) Artikel akan diterbitkan dalam jurnal yang didistribusikan ke seluruh dunia.

(4) Artikel, termasuk materi, dapat menjadi subjek siaran pers, dan dapat ditautkan ke / dari media sosial. Setelah diterbitkan, artikel akan ditempatkan di situs web penerbit dan mungkin juga tersedia di situs web pihak ketiga.

(5) Saya/pasien tidak akan menerima keuntungan finansial dari publikasi artikel.

(6) Saya dapat mencabut persetujuan saya kapan saja sebelum penulis menyerahkan artikel ke jurnal, tetapi setelah artikel tersebut berkomitmen untuk peer review, tidak akan mungkin untuk mencabut persetujuan.

(7) Formulir persetujuan ini akan disimpan dengan aman dan rahasia oleh Wiley, tidak lebih lama dari yang diperlukan.

Tanda tangan (Orang tua atau Wali)			
NAMA		Tanggal	
Hubungan dengan Pasien			

Rincian orang yang telah menjelaskan dan memberikan formulir kepada pasien atau perwakilan.

Tanda tangan (Penulis Utama)			
NAMA	Corry Quando Yahya	DATE	
ALAMAT	Jln. Palem Putri Raya No 17A, Palem Semi – Tangerang 15811		
EMAIL	Corry.spa@gmail.com		

Lampiran 4. Organisasi Peneliti

Peneliti utama : dr. Corry Quando Yahya, Sp.An-TI

Peneliti II : Dr. Kohar Hari Santoso, dr., SpAn-TI.,Subsp.An.Ped., Subsp TI

Lampiran 5. Lembar Penelitian

PERBANDINGAN KEJADIAN AGITASI ANESTESI INTRAVENA DEXMEDETOMIDINE DENGAN INHALASI SEVOFLURANE PADA OPERASI BIBIR SUMBING DAN CELAH LANGIT

A. Registrasi

No. Rekam Medis : Nomor Penelitian :

Hari / Tanggal operasi :

Diagnosis :

Prosedur pembedahan :

B. Identitas

1. Inisial Pasien : ______________________________
2. Tanggal Lahir (Umur) : ______________________________
3. Jenis Kelamin : L / P*
4. Tinggi Badan : _______cm
5. Berat Badan : _______kg
6. Diagnosis : ______________________________
7. ASA : I/II*
8. Penyulit ASA : ______________________________

C. Intra Operasi

1. Teknik Anestesia (Sevoflurane / Dexmedetomidine):____________________

2. Komplikasi Intra operasi :Bradikardia / Desaturasi / Laringospasme

3. Parameter Terpantau

Skala	Waktu (menit)													
	5'	10'	15'	20'	25'	30'	35'	40'	45'	50'	55'	60'	65'	70'
**Nadi /min**														
**Tekanan Darah (mmHg)**														
**Bispectral Index (BIS)**														
**Rescue Propofol (mg)**														

D. Pasca Operasi

1. Durasi Anestesi : _______ jam _______menit

2. Durasi Operasi : _______ jam _______menit

3. Waktu ekstubasi/ eksersi LMA :_________________menit

4. Waktu bangun/sadar penuh :_________________menit

5. Komplikasi Pasca operasi :Agitasi / Mual, muntah /Desaturasi / Laringospasme/ _____________________________

E. *Modified Yale Preoperative Anxiety Scale (mYPAS)*

	1	2	3	4	5	6	SKOR
**Kegiatan**	Melihat sekeliling, penasaran, bermain dengan mainan, membaca (atau perilaku sesuai usia lainnya); bergerak di sekitar area ruang tunggu untuk mendapatkan mainan atau pergi ke orang tua	Tidak menjelajah atau bermain, mungkin melihat ke bawah, mungkin gelisah dengan tangan atau mengisap ibu jari (selimut); mungkin duduk dekat dengan orang tua sambil menunggu	Berpindah dari mainan ke orang tua dengan cara yang tidak fokus; gerakan yang tidak diturunkan dari aktivitas; menggeliat, bergerak di atas meja, dapat mendorong masker menjauh atau menempel pada orang tua	Secara aktif mencoba melarikan diri, mendorong dengan kaki dan lengan, dapat menggerakkan seluruh tubuh; Di ruang tunggu, berlarian tidak fokus, tidak melihat mainan atau tidak akan berpisah dari orang tua			
**Vokalisasi**	Membaca (tidak vokalisasi sesuai dengan aktivitas), mengajukan pertanyaan, berkomentar, mengoceh, tertawa, siap menjawab pertanyaan tetapi mungkin umumnya tenang; Anak terlalu muda untuk berbicara dalam situasi sosial atau terlalu asyik bermain untuk merespons.	Menanggapi orang dewasa tetapi berbisik atau hanya menganggukan kepala	Tenang, tidak ada suara atau respons terhadap orang dewasa	Merintih, mengerang, mengerang, menangis diam-diam	Menangis atau mungkin berteriak "tidak"	Menangis, menjerit keras, berkelanjutan (terdengar melalui masker anestesi)	
**Ekspresi emosional**	Bahagia, tersenyum, atau berkonsentrasi pada permainan	Netral, tidak ada ekspresi yang terlihat di wajah	Khawatir (sedih) hingga mata ketakutan, sedih, khawatir, atau berkaca-kaca	Tertekan, menangis, sangat kesal, mungkin memiliki mata lebar			
**Kondisi keseluruhan**	Waspada, melihat sekeliling sesekali, memperhatikan apa yang dilakukan ahli anestesi dengannya dirinya	Ditarik, anak duduk diam dan tenang, mungkin mengisap ibu jari atau wajah berubah menjadi dewasa	Waspada, melihat sekeliling dengan cepat, mata lebar, tubuh tegang	Merintih panik, mungkin menangis atau mendorong orang lain menjauh, berpaling			

F. Skala Agitasi Cravero

Waktu (menit)									
	Extubasi	15'	30'	45'	60'	75'	90'	105'	120'
**Skala Cravero**									
Note									

Skala	Perilaku
1	Tidak memberikan respon pada stimulasi
2	Tertidur tenang. Anak memberikan respons saat diberikan stimulasi
3	Bangun dan memberikan respon kepada pengasuh
4	Menangis lebih dari 3 menit, tanpa perilaku kombatif
5	Menangis meraung, melakukan tindakan kombatif seperti mencabut infus, menendang dan tidak dapat ditenangkan oleh siapapun
1-3 = tidak delirium, 4-5 = delirium	

Lampiran 6. *Dummy Table*

Tabel 1.1 Karakteristik Subjek Penelitian

**Variabel**	**Semua Subjek**	**Sevoflurane**<br>**n**	**Dexmedetomidine**<br>**n**
Usia (dalam bulan)	Rerata ± s.d	Rerata ± s.d	Rerata ± s.d
Jenis Kelamin (Laki-laki/Perempuan)			
Berat Badan (kg)			
Klasifikasi ASA (I/II)			
Tipe Operasi (A/B)			
Lama Operasi (min)	Rerata ± s.d	Rerata ± s.d	Rerata ± s.d
Lama Anestesi (min)			
Komplikasi Intraoperasi (n/%)			
Komplikasi Pasca operasi (n/%)			

A = Bibir Sumbing, B = Celah langit

Tabel 1.2 Insiden agitasi (Skala Cravero >3)

**Kelompok**	**Jumlah (n)**	**Insidens (%)**	***Nilai p***
Sevoflurane			
Dexmedetomidine			

Tabel 1.3 Insiden komplikasi pasca operasi (Desaturasi, Laringospasme, Agitasi)

**Kelompok**	**Jumlah**	**Insidens (%)**	***Nilai p***
Sevoflurane			
Dexmedetomidine			

Tabel 1.5 Skala Cravero di Ruang Pemulihan (1-3 tidak agitasi, 4-5 agitasi)

**Waktu**	**Sevoflurane**	**Dexmedetomidine**	***Nilai p***
0 min			
15 min			
30 min			
45 min			
60 min			

Tabel 1.6 Perbandingan Waktu Pulih Sevoflurane dan Dexmedetomidine

**Kelompok**	**Durasi Anestesi (min)**	**Waktu Ekstubasi (min)**	**Waktu sadar (min)**	***Nilai p***
Sevoflurane				
Dexmedetomidine				

Lampiran 7. Rincian Biaya Penelitian

Judul Penelitian **:** Perbandingan Total Anestesi Intravena Dexmedetomidine dengan Inhalasi Sevoflurane pada Operasi Bibir Sumbing dan Celah Langit.

**1. Belanja barang habis pakai**					
**No**	**Deskripsi**	**Justifikasi pemakaian**	**Kuantitas**	**Harga satuan (Rp)**	**Biaya (Rp)**
1	Form Consent	Alat penelitian	100	Rp500	Rp50.000
2	Lembar pencatatan	Alat penelitian	100	Rp500	Rp50.000
3	Dexmedetomidine 200ug/vial	Obat penelitian	50	Rp166.500	Rp8.325.000
4	Spuit 20 cc	Alat penelitian	45	Rp3.500	Rp157.500
5	Perfusor tubing white	Alat penelitian	45	Rp80.000	Rp3.600.000
6	Three way connector	Alat penelitian	90	Rp12.000	Rp1.080.000
7	Needle 18G	Alat penelitian	20	Rp2.200	Rp44.000
8	NaCl 0.9% 100ml	Alat penelitian	20	Rp10.000	Rp200.000
9	Patient State Index monitor	Sewa alat penelitian	2	Rp1.000.000	Rp2.000.000
10	Sedline Probe	Alat penelitian	85	Rp500.000	Rp42.500.000
11	Syringe Pump	Sewa alat penelitian	2	Rp1.000.000	Rp2.000.000
				**Subtotal (Rp)**	Rp60.006.500

**2. Biaya Statistik**					
**No**	**Deskripsi**	**Justifikasi pemakaian**	**Kuantitas**	**Harga satuan (Rp)**	**Biaya (Rp)**
1	Konsultasi Statistik	Konsultasi dengan pembimbing statistik dan penyusunan ilmiah (Proposal dan Tesis)	2	Rp1.000.000	Rp2.000.000
				**Subtotal (Rp)**	Rp2.000.000

3. Biaya Operasional lain					
**No**	**Deskripsi**	**Justifikasi pemakaian**	**Kuantitas**	**Harga satuan (Rp)**	**Biaya (Rp)**
1	Kertas A4 80 gram	Biaya cetak proposal penelitian	1 rim	Rp60.000	Rp2.000.000
2	Fotokopi Proposal Penelitian	Biaya fotokopi bahan yang diserahkan kepada pembimbing dan dewan penguji	15 unit	Rp20.000	Rp300.000
3	Tinta Printer	Untuk mencetak Proposal	1 set	Rp80.000	Rp80.000
				**Subtotal (Rp)**	Rp2.380.000
