# Supplementary material for: Emergence agitation in pediatrics after dexmedetomidine vs. sevoflurane anesthesia: A randomized controlled trial
Source: PLoS One. 2025 Oct 8;20(10):e0333576. doi: 10.1371/journal.pone.0333576 (PMC12507210; doi:10.1371/journal.pone.0333576)
Supplement: Attachment — Submitted filename: Response to Reviewer 2.pdf [file pone.0333576.s006.pdf]

Reviewer #2:

Dear Reviewer,

We would like to thank you for your time and corrections on our manuscript. We appreciate your extensive review in order to make our manuscript clearer to the readers. Corrections on sentences or numbers are highlighted yellow on the text. Here is the answer to your questions:

1. Abstract, Line 38: "majority of the children receive" not "receives"

A1. Thankyou, we have made the necessary correction.

2. Line 43: better to say "intravenous DEX versus sevoflurane" otherwise the 'and' implies treatment with a combination of DEX and SEVO.

A2. Thankyou, we have made the necessary correction.

3. Results should include mean differences or odds/risk ratios for all outcomes, with 95% confidence intervals in addition to p-values, not p-values only. The primary outcome should be reported first. Small p-values should be reported as $p < 0.001$ not $p = 0.000$ which implies a zero p-value, which is incorrect.

A3. Thankyou, we have revised it to $p < 0.001$ (Line 57, 59, 234, Table 3, 259, Table 4.)

4. Introduction: line 74: "remains" rather than "remain". The end of that sentence could be shortened to "currently" instead of "at the current moment".

A4. Thankyou, we have changed the necessary correction at Line 79 and line 80.

5. Methods

A. All mentions of the numbers of children randomised (e.g. line 113) are results and should be reported in the results section.

A. Thankyou, we have moved it to the results section.

B. The sample size calculation is poorly reported. I would suggest: "The incidence of agitation among children anaesthetised with sevoflurane was estimated to be 40% [ref] and we hypothesised that a lower incidence of 10% would be observed with dexmedetomidine. We determined that a sample size of 35 patients in each group would give us 80% power to detect this absolute 30% difference using statistical testing at the 5% level of significance. Adjusting our sample size of anticipated 20% dropouts, we aimed to recruit 42 patients per group."

B. Thankyou, we have changed it as suggested. Line 170-174.

C. The statistical analysis is poorly reported. It is not clear exactly what was analysed by Kolmogrov-Smirnov (line 174). Generally, there is no role for these tests in a clinical trial analysis. It is also not clear what analysis of age and weight (line 175) were required, as none

of these are outcomes. Further, there is no role for any of the tests listed in line 177 to 179. This being a straightforward trial analysis, what were required were (1) a descriptive analysis of participant characteristics in each of the two groups according to baseline characteristics, with no statistical tests comparing the groups (this had been done in Table 1, although all reference to statistical tests should be removed, with no other changes), and (2) an analysis comparing outcomes between the groups, with mean (SE) for continuous outcomes or count (%) for binary outcomes in each arm, followed by unadjusted effects (mean difference, risk ratio, odds ratio etc) with 95% CI and p-value, and adjusted effects with 95%CI and p-value. If the parameters reported in Table 2 are considered outcomes, they should also be reported in this format, preferably with the other outcomes

C. Thankyou for your suggestion. We have deleted the information on equality tests for all descriptive data, as you suggested: Table 1 and Table 2.

Parameters reported in Table 2 are not considered outcomes as they are descriptive characteristics of what was found from the study. Hence, we have opted to remove the p-value out of Table 2.

The primary outcome from this study was incidence of emergence agitation after Sevoflurane versus Dexmedetomidine anesthesia. An analysis comparing both groups along with an odds ratio are mentioned in line 259-261. We have added another column on Table to include the OR and CI value. Table 4. Incidence of Emergence Agitation (Cravero Scale > 3)

6. **Results**

A. In the CONSORT diagram, reasons for exclusion should be reported

A. Thankyou, we have included a revised diagram for the exclusion reasons. Line 202-203

B. Throughout the results effects should be reported as mean differences or odds/risk ratios for all outcomes, with 95% confidence intervals in addition to p-values, not p-values only- see comments above regarding tables 1 to 3.

B. We have revised Table 4 to a revised version which includes the odds ratio and confidence interval of emergence agitation.
